# The IRF2/CENP-N/AKT signaling axis promotes proliferation, cell cycling and apoptosis resistance in nasopharyngeal carcinoma cells by increasing aerobic glycolysis

**DOI:** 10.1186/s13046-021-02191-3

**Published:** 2021-12-10

**Authors:** Cheng-Lin Qi, Mao-Ling Huang, You Zou, Rui Yang, Yang Jiang, Jian-Fei Sheng, Yong-Gang Kong, Ze-Zhang Tao, Hong-Yan Feng, Qing-Quan Hua, Li-Hong Bu, Shi-Ming Chen

**Affiliations:** 1 Department of Otolaryngology-Head and Neck Surgery, Renmin Hospital of Wuhan University, 238 Jie-Fang Road, Wuhan, Hubei 430060 P.R. China; 2 Institute of Otolaryngology-Head and Neck Surgery, Renmin Hospital of Wuhan University, 238 Jie-Fang Road, Wuhan, Hubei 430060 P.R. China; 3 PET-CT/MRI Center, Molecular Imaging Center, Renmin Hospital of Wuhan University, Wuhan, People’s Republic of China

**Keywords:** NPC, CENP-N, IRF2, AKT, Gglucose metabolism, Aerobic glycolysis

## Abstract

**Background:**

Centromere protein N (CENP-N) has been reported to be highly expressed in malignancies, but its role and mechanism in nasopharyngeal carcinoma (NPC) are unknown.

**Methods:**

Abnormal CENP-N expression from NPC microarrays of GEO database was analyzed. CENP-N expression level was confirmed in NPC tissues and cell lines. Stable CENP-N knockdown and overexpression NPC cell lines were established, and transcriptome sequencing after CENP-N knockdown was performed. In vitro and in vivo experiments were performed to test the impact of CENP-N knockdown in NPC cells. ChIP and dual luciferase reporter assays were used to verify the combination of IRF2 and CENP-N. Western blot analysis, cellular immunofluorescence, immunoprecipitation and GST pulldown assays were used to verify the combination of CENP-N and AKT.

**Results:**

CENP-N was confirmed to be aberrantly highly expressed in NPC tissues and cell lines and to be associated with high ^18^F-FDG uptake in cancer nests and poor patient prognosis. Transcriptome sequencing after CENP-N knockdown revealed that genes with altered expression were enriched in pathways related to glucose metabolism, cell cycle regulation. CENP-N knockdown inhibited glucose metabolism, cell proliferation, cell cycling and promoted apoptosis. IRF2 is a transcription factor for CENP-N and directly promotes CENP-N expression in NPC cells. CENP-N affects the glucose metabolism, proliferation, cell cycling and apoptosis of NPC cells in vitro and in vivo through the AKT pathway. CENP-N formed a complex with AKT in NPC cells. Both an AKT inhibitor (MK-2206) and a LDHA inhibitor (GSK2837808A) blocked the effect of CENP-N overexpression on NPC cells by promoting aerobic glycolysis, proliferation, cell cycling and apoptosis resistance.

**Conclusions:**

The IRF2/CENP-N/AKT axis promotes malignant biological behaviors in NPC cells by increasing aerobic glycolysis, and the IRF2/CENP-N/AKT signaling axis is expected to be a new target for NPC therapy.

**Supplementary Information:**

The online version contains supplementary material available at 10.1186/s13046-021-02191-3.

## Background

Head and neck squamous cell carcinoma (HNSCC) is a general term for cancers that occur in the anatomical areas of the nasal cavity, lips, tongue, oral cavity, pharynx, and larynx and arise from the malignant transformation of mucosal epithelium [1]. Nasopharyngeal carcinoma (NPC) is a subtype of HNSCC initiated by the malignant transformation of nasopharyngeal mucosal epithelial cells. In recent decades, with advances in treatment technology and changes in diet and lifestyle, the incidence and mortality rate of NPC have decreased significantly [2, 3]. It is even currently proposed that if the 5-year survival rate of patients with NPC can be increased to 95%, the treatment of NPC can be included in the management of chronic diseases [4]. However, there are still more unknown target genes and specific molecular biological mechanisms of action involved in regulating the development and progression of NPC; thus, a proportion of patients with NPC still have a poor prognosis when treated with combination therapy. Therefore, it is still important to conduct further in-depth investigations of the target genes and their molecular mechanisms that promote the development of NPC.

Centromere protein N (CENP-N), also called ICEN32, has an N-terminal region that recognizes CENP-A nucleosomes and a C-terminal region that binds CENP-L to form a mitophagy-associated network [5]. The CENP-N gene is located on chromosome 16q23.2, and the gene is widely found in eukaryotic chromosomal DNA [6]. The Japanese scholars Oka et al. demonstrated that CENP-N regulates the proliferation of oral squamous cell carcinoma through mediating the cell cycle [7]. Abnormal expression or posttranslational modification of proteins in the centromere protein (CENP) family can affect the proper assembly and function of kinetochores, which in turn leads to abnormal chromosome division and the production of micronuclei and is one of the most important factors in abnormal cell proliferation and division [8]. Abnormal cell proliferation and division is the most prominent genetic phenotype of malignant cells and an important intrinsic driver of the development and progression of malignancy. Unfortunately, no in-depth study on CENP-N and malignant tumorigenesis and progression has been conducted to date.

Interferon regulatory factor 2 (IRF2) is located on chromosome 4q35.1 and contains 12 exons. IRF2 competitively inhibits IRF1-mediated activation of the transcription of interferon α and β and other functional genes. IRF2 has been reported to inhibit the transcriptional activation of NFAT1 to regulate cell cycle progression in CD4+ T cells, influence cell differentiation and regulate apoptosis [9]. In addition, Mashima et al. found that IRF2 is involved in regulating SNARE-mediated paracrine secretion in pancreatic cells [10]. Some studies have shown that the IRF2-INPP4B signaling axis is involved in regulating the proliferation and survival of acute myeloid leukemia cells [11]. However, no studies on the ability of IRF2 to regulate CENP-N have been reported.

Protein kinase B (AKT) is a core member of the PI3K/AKT signaling pathway. AKT can be widely involved in the development and progression of almost all malignancies, including NPC [12–14]. In malignant cells, AKT is involved in the regulation of downstream effector protein molecules that are associated with various malignant features of cancer cells, such as high glucose metabolism, proliferation, angiogenesis and invasive metastasis [13, 15]. The AKTs identified to date mainly include three isoforms, AKT1 (PKBα), AKT2 (PKBβ) and AKT3 (PKBγ), which are highly similar in structure but differ slightly in the physiological functions they are involved in: AKT1 mainly mediates cell proliferation and cell cycle regulation, AKT2 primarily regulates cell migration, while AKT3 is involved in regulating the resistance of malignant tumor cells to chemotherapeutic agents [16, 17]. However, due to the high structural similarity of AKT proteins, the regulation of cell biological functions by the three types of AKT proteins also intersects to some extent.

Here, we first identified and validated CENP-N as an oncogene associated with the promotion and progression of nasopharyngeal carcinogenesis. We sought to explore the transcription factors that influence changes in CENP-N gene expression in NPC. Our results revealed that IRF2 bound to two sites in the promoter region of CENP-N and that promoted the transcription of CENP-N. We further explored the specific molecular mechanisms by which CENP-N promotes the malignant biological behavior of NPC cells. The results indicated that the down-regulation of CENP-N significantly inhibited AKT phosphorylation at Ser473, leading to the inhibition of AKT activation. Finally, we found the IRF2/CENP-N/AKT axis promotes malignant biological behaviors in NPC cells by increasing aerobic glycolysis, the IRF2/CENP-N/AKT signaling axis is expected to be a new target for NPC therapy.

## Materials and methods

### Bioinformatic analysis

Differentially expressed genes (DEGs) were analyzed in the GSE12452 and GSE53819 gene sets using |log_2_FC| ≥ 1 and adj. *P* < 0.05 as screening criteria. A PPI network was constructed via String database analysis and visualized using Cytoscape [18]. Survival prognosis was analyzed using the Kaplan-Meier plotter database and the GEPIA database. CENP-N transcription factor predictions were obtained from the GeneCards database, PROMO database and JASPAR database. The links to the databases used are provided in Supplementary Table 1.

### Cell culture and tissue specimens

NPC cell lines (CEN-2Z, SUNE-1, 5-8F, 6-10B, and HK-1) were cultured in DMEM (Gibco, MD, USA) medium containing 1% penicillin-streptomycin and 10% FBS (Gibco, MD, USA) at 5% CO_2_ and 37 °C. NP460 cells were cultured in growth factor-containing keratinocyte serum-free medium (KSFM) [19].

Tissue microarrays (TMA, NO.1501, NO.131 and NO.132) were provided by Fanpu Biotech (Guilin, China). NO.1501 and NO.131 were used for CENP-N analysis. NO.132 was used for IRF2 detection. Imaging data for 21 NPC patients who underwent PET/CT imaging at the PET Imaging Center of the People’s Hospital of Wuhan University were selected, and paraffin tissue sections of the corresponding patients were obtained through the Department of Pathology of the People’s Hospital of Wuhan University (Supplementary Table 2). In addition, we collected 20 fresh NPC specimens between January 2019 and October 2020.

### Immunofluorescence assay

Tissue sections were blocked for 1 h using 10% BSA after baking, dewaxing and hydration, and antigen retrieval steps. The sections were then incubated overnight with an primary antibody. The next day, the sections were incubated with a FITC-labeled or Cy3-labeled secondary antibody for 1 h under protection from light, dried, and sealed with anti-fluorescence quenching solution, and fields of view were then selected under a fluorescence microscope for observation and image acquisition. An informative list of the antibodies was shown in Supplementary Table 3.

### Immunohistochemical staining analysis

Tissue samples were processed and analyzed according to a previous article [20, 21]. In this study, we assigned samples with negative and weakly positive immunohistochemical scores to the CENP-N low expression group and samples with positive and strongly positive scores to the CENP-N high expression group. An informative list of the antibodies was shown in Supplementary Table 3.

### Generation of knockdown cells

Detailed steps are provided in a previous article [22]. Transfected cells were subsequently selected using appropriate concentrations (1–5 μg/mL) of puromycin and were then mass passaged and frozen for seeding and subsequent cell experiments.

### qRT-PCR assay

Total cellular RNA was extracted using TRIzol and was then reverse transcribed to cDNA. qRT-PCR was subsequently performed using a SYBR Green qPCR Kit (Takara, Mountain View, CA) according to the procedure. β-actin was used as the internal reference. The relative mRNA expression levels of target genes were calculated by the 2^-ΔΔCT^ method. Primer sequences are shown in Supplementary Table 4.

### RNA-seq data analysis

KEGG is a public database containing annotations for intrinsic associations of multiple gene functions [23]. CENP-N knockdown NPC cells and control NPC cells were delivered to Crystal Energy Biotechnology (Shanghai) Co., Ltd. (https://www.genenergy.cn/) for high-throughput transcriptome sequencing of multiple cell samples using the Illumina HiSeq sequencing platform. The sequence alignment results for each group of samples were further screened for differentially expressed genes (DEGs) that showed changes after treatment using DESeq2 software, with |log2FC|≧1 and *P* < 0.05 defined as the screening criteria for DEGs. Finally, the obtained DEGs were incorporated into the KEGG and GO database for further enrichment analysis.

### Western blot analysis and reagents

Western blot assay was performed as previously described [24]. The PVDF membrane were purchased from Millipore (MA, USA). An informative list of the antibodies was shown in Supplementary Table 3.

MK-2206, 2-DG and GSK2837808A, used in the study, are a chemically synthesized AKT inhibitor, a glucose uptake inhibitor and a lactate dehydrogenase A (LDHA) inhibitor, respectively (all purchased from MedChemExpress, NJ, USA). The working concentration of MK-2206 was 10 μmol/L [25]. The working concentration of 2-DG was 2 mmol/L [26]. The working concentration of GSK2837808A was 10 μmol/L [27].

### Glucose uptake assay and lactate determination

Cells were incubated in pyruvate-free DMEM (Gibco, MD, USA) for 6 h prior to glucose uptake and lactate determination assays [27]. Then, the cells were processed and analyzed as previously described [28].

Glucose uptake assay: The absorbance value of each sample at 505 nm was measured using an enzyme marker. Final sample cellular glucose uptake content = medium glucose content - sample glucose content.

Lactate determination: The absorbance value of each sample at 530 nm was measured using an enzyme standard. Final sample lactate concentration = (sample assay OD - blank assay OD)/(standard assay OD - blank assay OD) × sample concentration [28].

### CCK-8 and Colony formation assays

Cell Counting Kit-8 (CCK-8, Dojindo, Shanghai, China) and clonogenic assays were used to evaluate cell survival and proliferation. Cells were processed and analyzed as previously described [24].

### Flow cytometric analysis of apoptosis and the cell cycle

Detailed steps are provided in a previous article [7, 24]. The data were analyzed using FlowJo 7.6 software.

### Construction of an NPC tumor model in nude mice and PET/CT imaging of small animals

Twenty-four 4-week-old female BALB/c nude mice (Huafukang Biotechnology Co., Beijing, China) were housed in the SPF environment of the Animal Experiment Center of the People’s Hospital of Wuhan University.

Detailed steps for model establishment are provided in a previous article [29] The tumor volume was measured every day after successful model establishment. The final tumor volume (V) was calculated as follows: V = (a × b × b)/2 [30].

When the average maximum diameter of the tumors in a group of nude mice was ≥1.0 cm, small animal PET/CT imaging was performed (Raydata Technology Co., Wuhan, China). The maximum standardized uptake value (SUVmax), the mean standardized uptake value (SUVmean) and total lesion glycolysis (TLG) were analyzed. Tumor tissues for subsequent experiments were harvested from nude mice under adequate anesthesia [31].

### Chromatin immunoprecipitation assay

The experiment was carried out according to a previous description [22]. The primer sequences are shown in Supplementary Table 5. The input group was used as a control for each group of results, and the data from the IgG(anti- IgG antibody) and IP(anti-IRF2 ChIP antibody) groups were compared with those from the input group and were then analyzed to compare whether there was a change in the amount of enriched DNA of the transcription factor IRF2 between the IP and IgG groups. An informative list of the antibodies was shown in Supplementary Table 3.

### Luciferase reporter assay

The dual luciferase reporter assay was performed as described in a previous study [22]. The cloned human IRF2 gene was first inserted into the vector pcDNA3.1 (pCDNA3.1-IRF2). Subsequently, two promoter sequences of human CENP-N were inserted into the vector pGL3-Basic (labeled pGL3- CENPN-1 and pGL3- CENPN-2). The constructed plasmids and the pRL-TK plasmid containing the sequence of Renilla (sea pansy) luciferase were transfected into 5-8F and CNE-2Z cells. Cells were grown to the appropriate density and were then lysed, and the lysates were collected. Subsequently, firefly luciferase assay reagent and Renilla luciferase assay reagent were added to the machine for detection. Finally, the ratio of firefly luciferase intensity (RLU-1) to Renilla luciferase intensity (i.e., RLU-1/RLU-2) was used as an internal reference for the activation of target genes in each sample to be tested.

### Cellular immunofluorescence colocalization assay

Cells were fixed for 30 min, and an anti-CENP-N primary antibody (1:100) and anti-AKT primary antibody (1:100) were then added dropwise and incubated overnight at 4 °C. The following day, the slides were incubated with the FITC-labeled goat anti-mouse secondary antibody (1:100) for 1 h and then with the CY3-labeled goat anti-rabbit secondary antibody (1:300) for 1 h. Subsequently, nuclei were stained with DAPI for 15 min at room temperature, and the slides were then sealed with anti-fluorescence quenching solution. Staining of cell crawling was observed, and images were acquired using a confocal microscopy (Olympus, Tokyo, Japan) to select the appropriate field of view. Then the images were analyzed with ImageJ (W S Rasband, Image J, NIH, USA) [27]. An informative list of the antibodies was shown in Supplementary Table 3.

### Co-IP assay

The procedure for the cellular protein immunoprecipitation assay was described in a previous study [32]. Cells were collected and lysed in RIPA lysis buffer (Beyotime) containing a protease inhibitor cocktail (Roche Diagnostics). Whole-cell lysates (2 mg) were precleared with 30 μL of protein G beads (Life Technologies), and 2 μg of isotype-matched IgG control or the indicated antibodies were then added and incubated for 2 h on a rocking platform. Immunoprecipitates were collected by centrifugation and were then resolved by SDS-PAGE. An informative list of the antibodies was shown in Supplementary Table 3.

### GST pulldown assay

The GST pulldown assay procedure was performed as described in a previous study [33]. The GST-CENP-N and His-AKT1 genes were first cloned and synthesized and were then inserted into pGEX-6P-1. After insert ligation, transformation into recipient cells and clonal expression, protein samples containing 500 μg of GST (control group) or GST-CENP-N (experimental group) were added to glutathione-agarose resin after 3 h of mixing. Both the control and experimental groups were mixed overnight after adding 500 μg of His-AKT1 protein. The following day, the samples of both groups were centrifuged, the appropriate amount of protein loading buffer was added, and the samples were then incubated in a 100 °C water bath for 5 min. Finally, GST/GST-CENP-N and His-AKT1 were subjected to SDS-PAGE and immunoblotting with an anti-GST antibody and an anti-His antibody, respectively, to detect whether the decoy protein CENP-N can directly bind to the target protein AKT1.

### Statistical analysis

All data collected in this study were analyzed using the medical statistics software SPSS 20.0, and the data are expressed as the mean ± standard deviation (^−^χ ± s) values. Comparisons of two independent samples were performed using a t-test; survival analysis was performed using the Kaplan-Meier method, and statistical analysis of survival data was performed using the log-rank test. GraphPad Prism 8 was used in this study to generate relevant statistical plots. For the purpose of this study, *P* < 0.05 was considered to indicate a statistically significant difference.

## Results

### CENP-N was significantly upregulated in NPC and was related to high glucose metabolism and poor prognosis

To investigate the role of gene expression abnormalities in the development and progression of NPC, we selected two sequencing microarrays from the GEO database for DEGs analysis. The results showed that 1280 DEGs, specifically, 731 downregulated and 549 upregulated genes, were identified in the GSE12452 chip; 2389 DEGs, specifically, 910 upregulated and 1479 downregulated genes, were identified in the GSE53819 chip (Fig. S1A). Subsequently, we selected the DEGs common to both chips and finally identified 207 upregulated genes and 361 downregulated genes (Fig. S1B). Finally, these DEGs were subjected to PPI network analysis and visualization, and 37 candidate hub genes differentially expressed in NPC were further identified based on their protein interactions (Fig. S1C).

We imported these 37 genes into the Kaplan-Meier plotter database and further identified 20 candidate hub genes associated with the survival prognosis of HNSCC patients (Fig. S1D). Subsequently, these 20 genes were imported into the GEPIA database, and three differentially expressed hub genes (Fig. S1E) were finally identified according to the relationship between gene expression and disease-free survival of HNSCC patients. In addition, we analyzed the expression levels of CENP-N in different tissues/organs and tumors based on the information provided in the GENE and GEPIA databases, respectively (Fig. S1F-G).

**Fig. 1:**
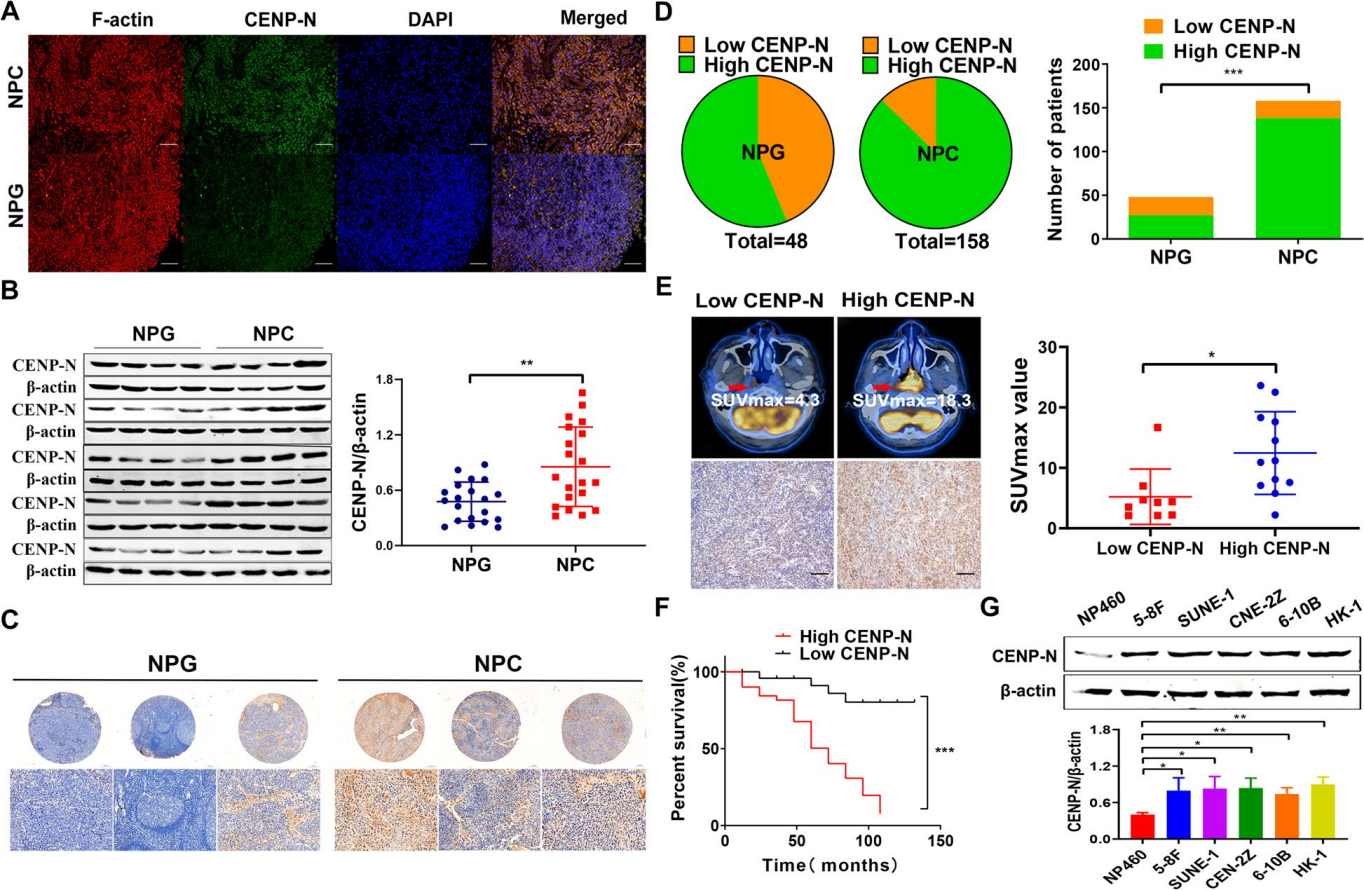
CENP-N expression in NPC tissues and cells. **a** Representative images from NPC and NPG tissues with F-actin and CENP-N immunofluorescence staining (200×). **b** The expression of CENP-N in NPC and NPG tissues was evaluated by Western blotting, and the results showed that CENP-N was highly expressed in NPC cells. **c** Representative images from NPC and NPG tissue samples with low or high CENP-N immunohistochemical staining scores (4× and 200×). **d** Statistical analysis of the immunohistochemical results showed that CENP-N was highly expressed in NPC. **e** Representative ^18^F-FDG PET-CT images and SUVmax of NPC samples from NPC patients with low or high CENP-N expression (all 200×). **f** Survival curve of patients stratified by CENP-N expression. **g** Expression of CENP-N in NPC and NP460 cell lines. The data are shown as the mean ± SD values. * *p* < 0.05, ** *p* < 0.01, ***p < 0.001

Subsequently, we found that the CENP-N expression was elevated in NPC tissues compared with NPG by using immunofluorescence staining and Western blot analysis (Fig. 1a-b). To further investigate the expression level of CENP-N in NPC, we used a large sample of NPC patients with TMA immunohistochemical staining scores, and the immunohistochemical scores were analyzed with the chi-square test: CENP-N expression was elevated in NPC tissues, and the difference was statistically significant (Fig. 1c-d and Supplementary Table 6, *P* < 0.05).

Then, we analyzed PET/CT imaging data of 21 patients with NPC and the immunohistochemical staining results of the corresponding pathological tissue sections and found that tumor glucose metabolism was increased in the primary lesions of patients with high expression of CENP-N in NPC tissues compared with those of patients with low expression (Fig. 1e and Supplementary Table 2, *P* < 0.05).

Furthermore, we analyzed the immunohistochemical staining results of NPC tissue microarrays containing samples from 98 patients with 10-year follow-up data and found that high expression levels of CENP-N were associated with poor survival prognosis in NPC patients (Fig. 1f, *P* < 0.05).

Finally, the expression of CENP-N in the NPC cell lines 5-8F, SUNE-1, CNE-2Z, 6-10B and HK-1 was analyzed. The results confirmed that CENP-N was significantly high expression in several NPC cell lines (Fig. 1g, *P* < 0.05).

### CENP-N significantly affected glucose metabolism, cell cycling, cell proliferation, and apoptosis-related RNA and protein expression in NPC cells

**Fig. 2:**
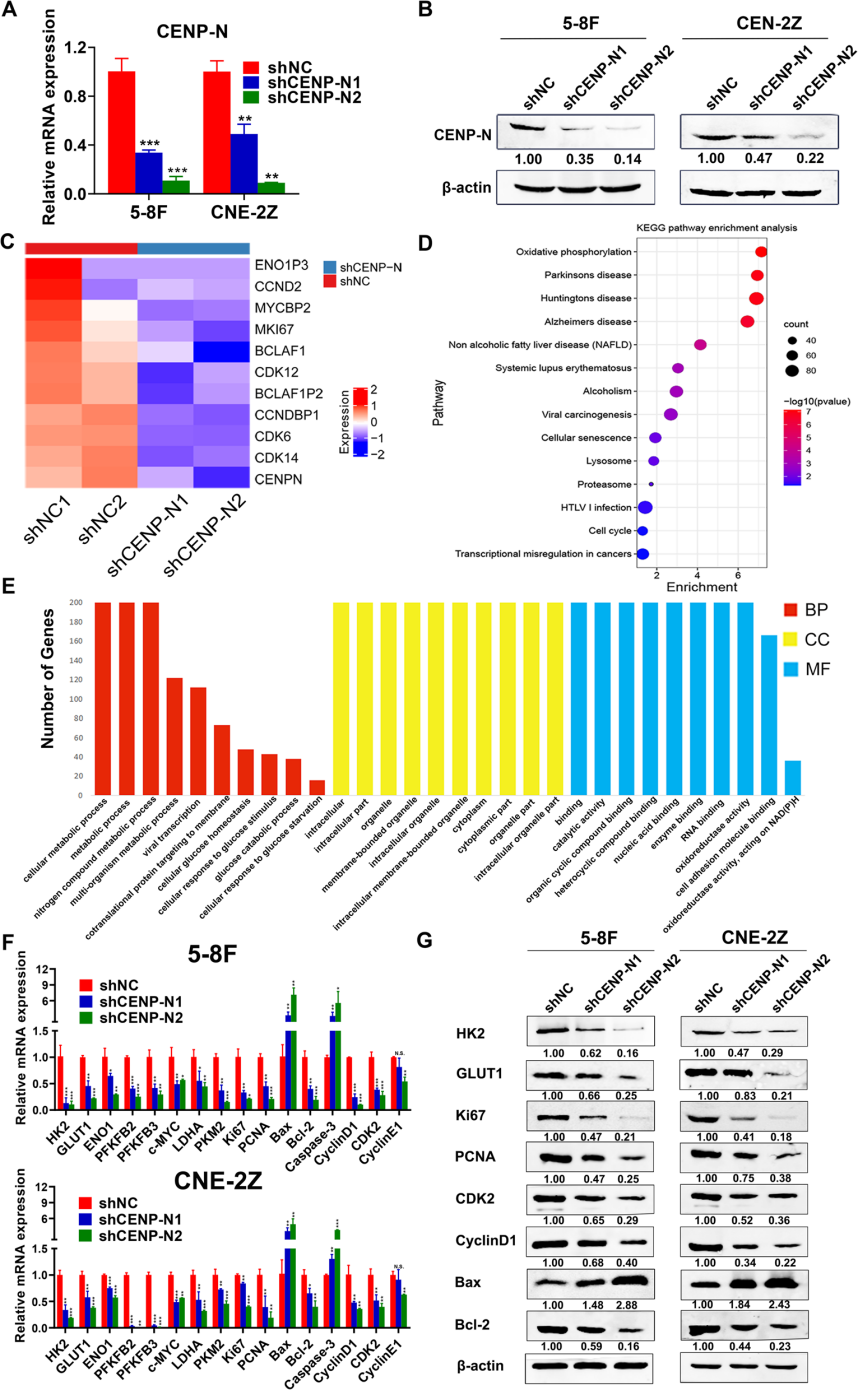
RNA-sequencing after downregulating CENP-N. **a** qRT-PCR was used to verify CENP-N knockdown in 5-8F and CNE-2Z cell lines. **b** Western blotting was used to verify CENP-N knockdown in 5-8F and CNE-2Z cells. **c** Heat map showing that the genes altered after CENP-N knockdown were involved in glycolysis, apoptosis, proliferation, and cell cycling. **d** Bubble plot of KEGG pathway analysis results showing the function of genes altered after CENP-N knockdown. **e** Bar plot of GO analysis results showing the function of genes altered after CENP-N knockdown. **f** qRT-PCR was used to verify genes altered after CENP-N knockdown involved in cell glycolysis, apoptosis, proliferation, and cell cycle progression. **g** Western blotting was used to verify genes altered after CENP-N knockdown involved in glycolysis, apoptosis, proliferation, and cell cycling. The data are shown as the mean ± SD values. * *p* < 0.05, ** *p* < 0.01, ***p < 0.001

To further investigate the role of CENP-N in nasopharyngeal carcinogenesis and development, we generated CENP-N knockdown cell lines (shCENP-N1 and shCENP-N2) with two shRNA sequences in the NPC cell lines 5-8F and CNE-2Z (Fig. 2a-b, *P* < 0.05).

Subsequently, we sequenced the transcriptome of the CNE-2Z after knocking down CENP-N and found by pathway enrichment and GO analysis that the gene transcriptome expression changes in NPC cells predominantly occurred in genes and cell signaling pathways related to glucose metabolism, cellular senescence, and cell cycle progression (Fig. 2c-e).

We further validated the effect of knockdown of CENP-N on glucose metabolism, cell proliferation, cell cycling and apoptosis in NPC cells by qRT-PCR (Fig. 2f). The results confirmed that knockdown of CENP-N resulted in downregulation of HK2, GLUT1, ENO1, PFKFB2, PFKFB3, LDHA, PKM2 and c-MYC in both NPC cell lines. In addition, we examined the effect of CENP-N knockdown on the protein expression levels of HK2, GLUT1, Ki67, PCNA, CDK2, CyclinD1, Bax and Bcl-2 in NPC cells (Fig. 2g). The results confirmed that knockdown of CENP-N resulted in downregulation of HK2, GLUT1, Ki67, PCNA, CDK2, CyclinD1 and Bcl-2 expression and upregulation of Bax expression in both NPC cell lines. Gnens like HK2 and GLUT1 are involved in both aerobic and anaerobic glucose metabolism. Gnens like ENO1, PFKFB2, PFKFB3, LDHA, PKM2 and c-MYC are involved in aerobic glycolysis [22, 27, 34]. These results confirmed that knockdown of CENP-N can affect the key genes involved in aerobic glycolysis, cell proliferation, cell cycling and apoptosis in NPC cells under in vitro conditions.

### CENP-N knockdown in NPC cells resulted in a significant decrease in glucose metabolism, proliferation, cell cycling and apoptosis resistance

**Fig. 3:**
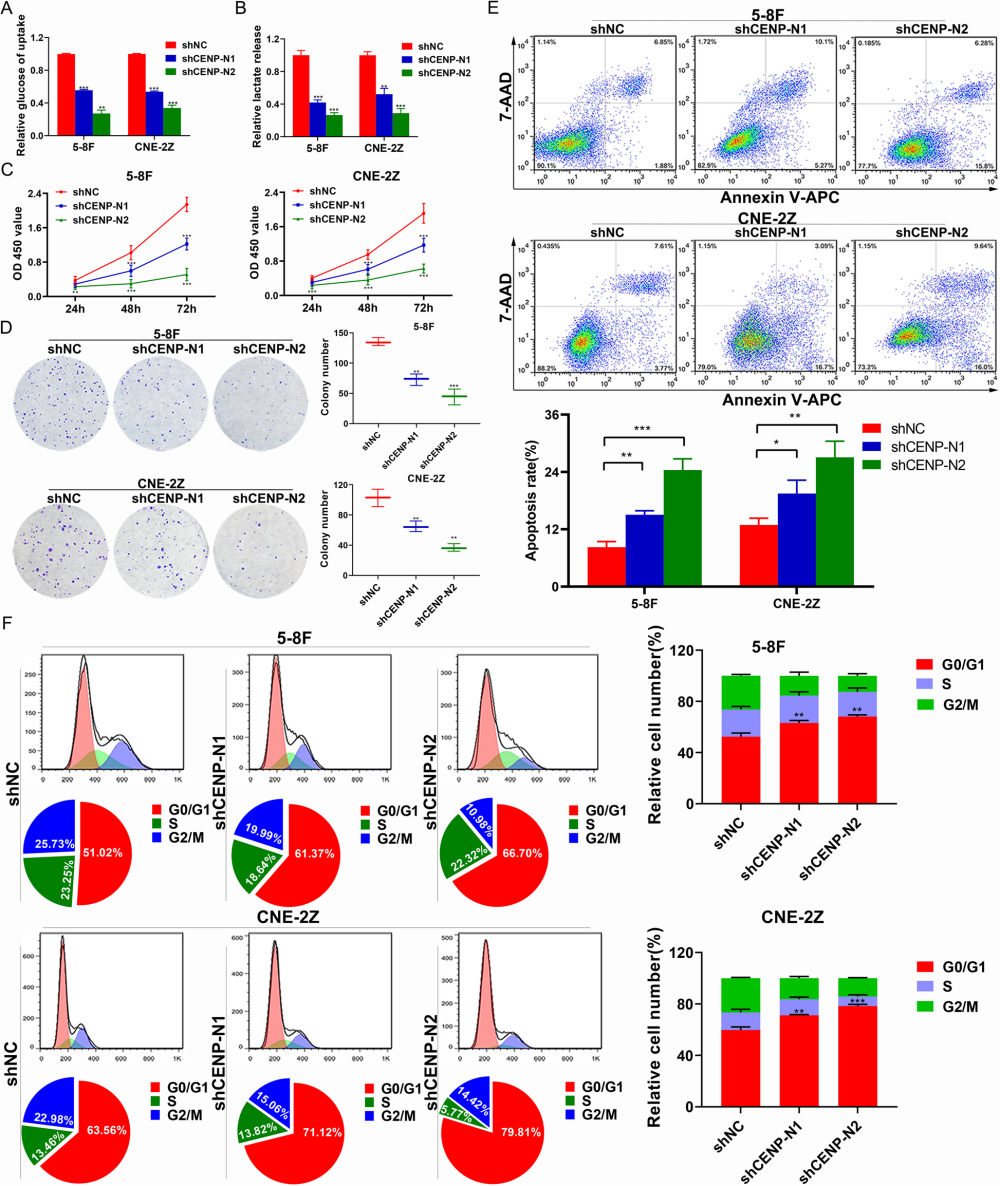
Changes in malignant biological behaviors of two NPC cell lines after downregulation of CENP-N. **a** Changes in relative cellular glucose uptake detected after knockdown of CENP-N in two NPC cell lines. **b** Changes in cellular lactate production detected after knockdown of CENP-N in two NPC cell lines. **c** Changes in cell viability detected by a CCK-8 assay after knockdown of CENP-N in two NPC cell lines. **d** Changes in cell proliferation detected after knockdown of CENP-N in two NPC cell lines. **e** Changes in the percentage of apoptotic cells detected after knockdown of CENP-N in two NPC cell lines. **f** Changes in the cell cycle distribution after knockdown of CENP-N in 5-8F and CNE-2Z cell lines. The data are shown as the mean ± SD values. * *p* < 0.05, ** *p* < 0.01, ***p < 0.001

To investigate the effect of CENP-N on malignant biological behaviors in NPC cell lines, we designed 2 sequences to knock down CENP-N and subsequently assayed its effect on malignant biological behaviors of cells in vitro. The results showed that both NPC cell lines exhibited reduced glucose uptake and decreased lactate production after knockdown of CENP-N relative to the control group (Fig. 3a-b, *P* < 0.05); both NPC cell lines exhibited reduced cell survival and proliferation after knockdown of CENP-N, and the differences were statistically significant (Fig. 3c-d, *P* < 0.05); both NPC cell lines showed an increase in the apoptosis rate after knockdown of CENP-N, and the difference was statistically significant (Fig. 3e, *P* < 0.05); and both NPC cell lines showed G0/G1 cell cycle arrest after knockdown of CENP-N (Fig. 3f).

### Knockdown of CENP-N suppresses NPC progression by altering glucose metabolism, cell proliferation, cell cycling and apoptosis in vivo

**Fig. 4:**
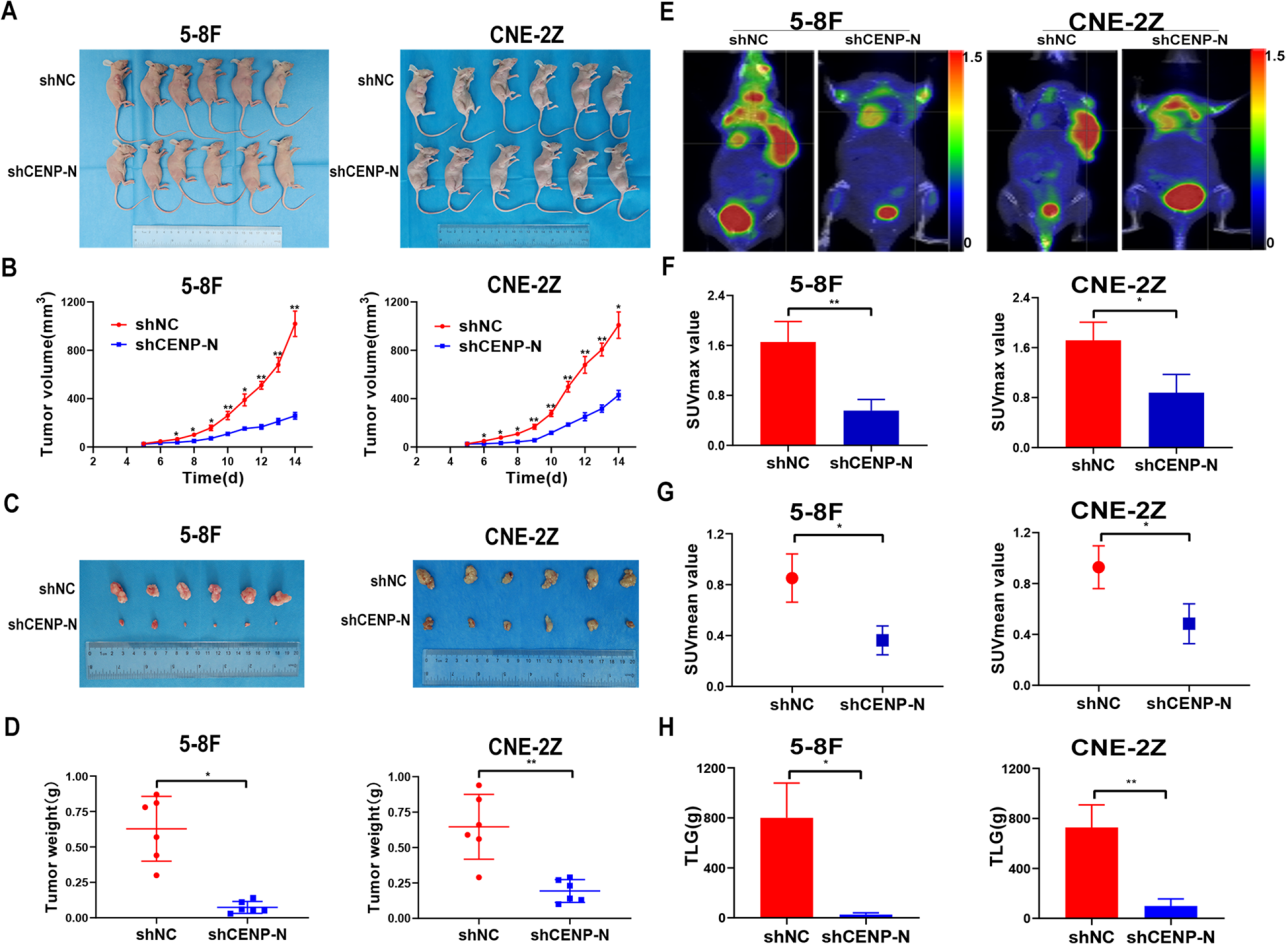
Downregulation of CENP-N inhibited cell proliferation and glucose metabolism in vivo. **a** General view of tumor formation in nude mice. **b** The tumor volume curve of nude mice bearing tumors formed from 5-8F shCENP-N cells, CEN-2Z shCENP-N cells and the corresponding control cells. **c** Nude mice bearing tumors formed from 5-8F shCENP-N cells, CEN-2Z shCENP-N cells and the corresponding control cells. **d** The average tumor weight in nude mice bearing tumors formed from 5-8F shCENP-N cells, CEN-2Z shCENP-N cells and the corresponding control cells. **e** A representative PET-CT image was used to detect glucose uptake in 5-8F shCENP-N and CEN-2Z shCENP-N xenografts and the corresponding controls. **f** SUVmax values in nude mice bearing tumors. **g** SUVmean values in nude mice bearing tumors. **h** TLG values in nude mice bearing tumors. The data are shown as the mean ± SD values. * *p* < 0.05, ** *p* < 0.01

To investigate the effect of CENP-N knockdown on biological behaviors of NPC cells in vivo, we first established an NPC xenograft tumor model in nude mice using shNC cells as the control cells and shCENP-N2 cells as the experimental cells (Fig. 4a). Compared with mice in the control group implanted with either NPC cell line, mice exhibited significantly slower tumor growth (Fig. 4b) and reduced tumor weight (Fig. 4c-d) after knockdown of CENP-N in vivo (*P* < 0.05). These results confirmed that knockdown of CENP-N significantly inhibited the malignant proliferation of NPC cells in vivo. In addition, we performed small animal PET/CT imaging on nude mice in the xenograft tumor models of both NPC cell lines (Fig. 4e). The results showed that relative to shNC tumors, tumors formed from both shCENP-N NPC cell lines exhibited a reduced maximum standardized glucose uptake (Fig. 4f, a reduced mean glucose uptake (Fig. 4g) and reduced total glycolysis (Fig. 4h). These results confirmed that knockdown of CENP-N significantly inhibited glucose metabolism in NPC cells in vivo.

**Fig. 5:**
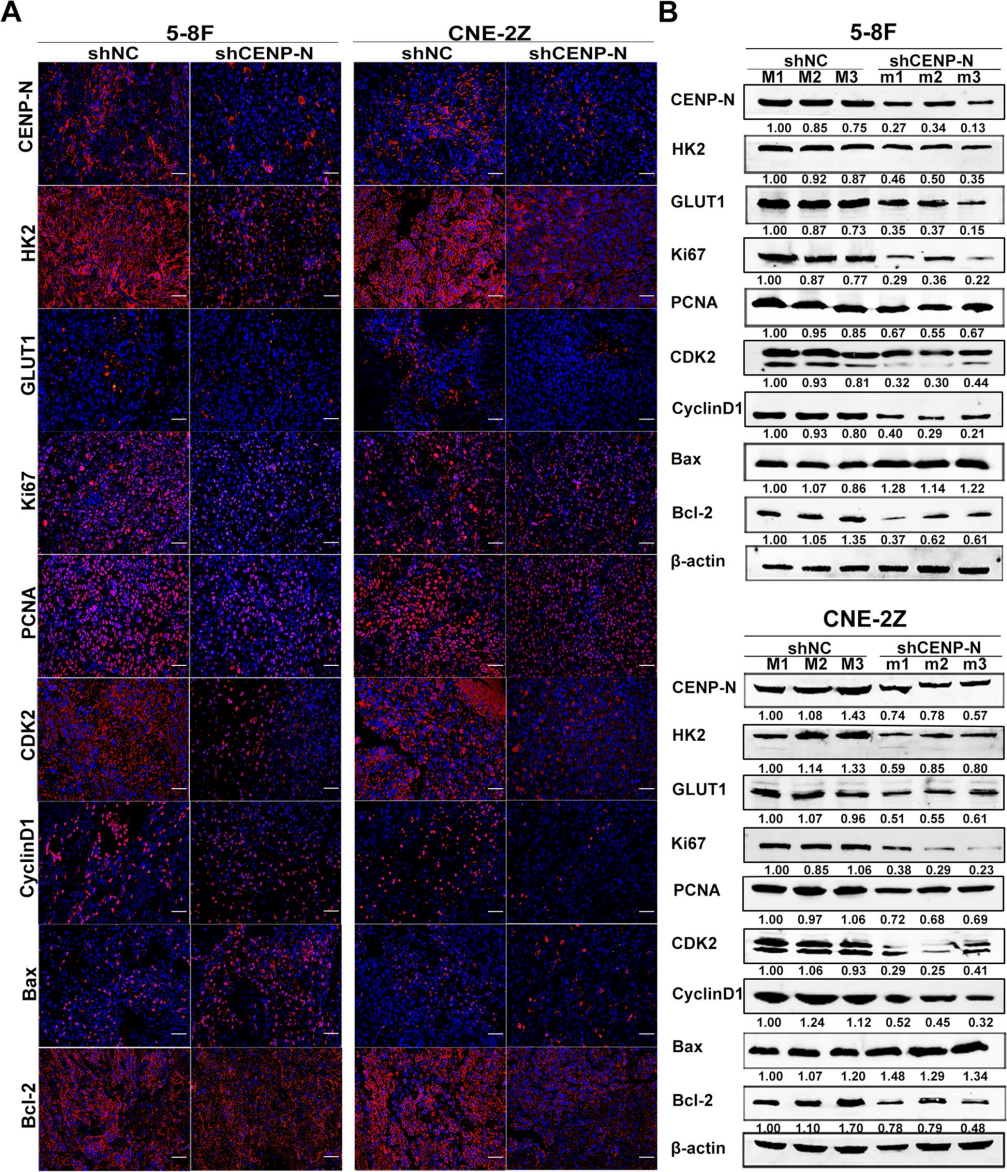
Expression levels of proteins related to cell proliferation, cell cycling, glucose metabolism and apoptosis were measured in xenografts formed from cells with CENP-N knockdown. **a** Immunofluorescence staining showed the expression of proteins related to cell proliferation, cell cycling, glycolysis and apoptosis in 5-8F shCENP-N and CEN-2Z shCENP-N xenografts and the corresponding controls (scale bar, 50 μm). **b** Western blot analysis showing the expression of proteins related to cell proliferation, cell cycling, glycolysis and apoptosis in 5-8F shCENP-N and CEN-2Z shCENP-N xenografts and the corresponding controls

Finally, we performed HE staining (Fig. S2A), immunofluorescence staining (Fig. 5a), immunohistochemical staining (Fig. S2B-C), and Western blot analysis (Fig. 5b) on nude mouse tumor tissues to detect protein expression changes in HK2, GLUT1, Ki67, PCNA, CDK2, CyclinD1, Bax, and Bcl-2. The results showed that the expression of HK2, GLUT1, Ki67, PCNA, CDK2, CyclinD1 and Bcl-2 was downregulated, while Bax expression was upregulated after knockdown of CENP-N in two NPC cell lines in vivo (*P* < 0.05). These results confirm that knockdown of CENP-N can also suppress NPC progression by altering the expression of proteins involved in glucose metabolism, cell proliferation, cell cycling and apoptosis in vivo.

### IRF2 regulated CENP-N at the transcriptional level in NPC cells

**Fig. 6:**
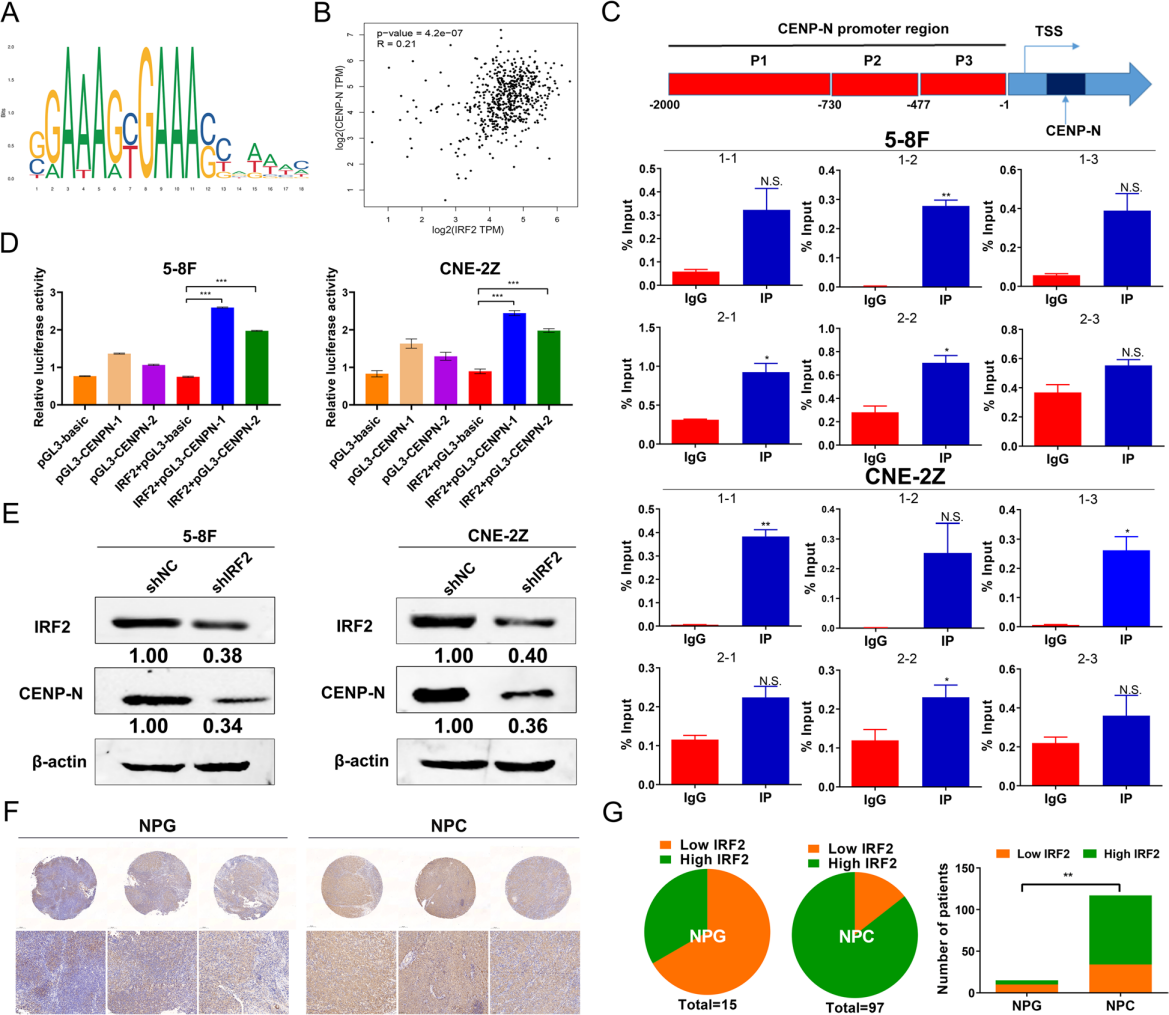
CENP-N was regulated directly by IRF2 in NPC cells. **a** Sequence logo of the IRF2 binding site in the CENP-N promoter. **b** The GEPIA website was used to predict the correlation between IRF2 and CENP-N expression. **c** PCR analysis of the ChIP product showed that IRF2 bound to the CENP-N gene promoter region. **d** A luciferase reporter assay was used to detect the effects of IRF2 binding site in the promoter region of CENP-N. **e** Western blotting was used to verify the correlation between IRF2 and CENP-N expression. **f** Representative images from NPG and NPC tissue samples with low or high IRF2 immunohistochemical staining scores (4× and 200×). **g** Statistical analysis of the immunohistochemical results showed that IRF2 was highly expressed in NPC. The data are shown as the mean ± SD values. * *p* < 0.05, ** *p* < 0.01, ***p < 0.001

We sought to explore the transcription factors that influence changes in CENP-N gene expression in NPC. Firstly, 311 transcription factors were screened by GeneCards database. And then 30 transcription factors were screened by PROMO database. Combined with venn diagram, five transcription factors (IRF2, YY1, WT1, ATF2 and ELK1) were shared as the transcription factors of CENP-N (Fig. S3A). Finally, only IRF2 was verified as the transcription factors of CENP-N by JASPAR and GEPIA database. The results revealed that there were binding sites for IRF2 in the CENP-N promoter sequence (Fig. 6a). The GEPIA database prediction revealed that the expression of IRF2 was positively correlated with that of CENP-N (Fig. 6b). These results suggested that IRF2 may be a transcriptional regulator of CENP-N. Moreover, the ChIP-PCR results revealed that IRF2 was able to bind to 2 sites in the promoter region of CENP-N (Fig. 6c). This result confirmed that IRF2 can bind to the promoter sequence of CENP-N and may be a transcription factor of CENP-N.

The dual luciferase reporter assays results showed that the relative luciferase activities in both IRF2 + pGL3-CENP-N-1 cotransfected cells and IRF2 + pGL3-CENP-N-2 cotransfected cells were significantly enhanced relative to that in IRF2 + pGL3-Basic cotransfected cells (Fig. 6d). These results confirmed that IRF2 bound to two sites in the promoter region of CENP-N and that both binding events promoted the transcription of CENP-N.

In addition, it was found that CENP-N protein expression was significantly downregulated in the shIRF2 group relative to the shNC group in the NPC cell lines (Fig. 6e). This result confirmed that IRF2 downregulation significantly suppressed CENP-N expression. Finally, IRF2 was confirmed to be aberrantly highly expressed in NPC tissues (Fig. 6f-g) and cell lines (Fig. S3B).

To investigate whether knockdown of CENP-N in 5-8F and CNE-2Z cells can block the oncogenic effect of IRF2 on NPC cells, we established three groups of NPC cells—shNC, shIRF2 and shIRF2 + oeCENP-N—and examined the changes in glucose uptake and lactate production, cell proliferation, apoptosis and cell cycling in the three groups of cells. The results confirmed that IRF2 knockdown produce inhibitory effect on the glucose metabolism that can be reverted by introducing CENP-N in NPC cells (Fig. S3C-D). IRF2 knockdown produce inhibitory effect on the survival and proliferation that can be reverted by introducing CENP-N in NPC cells (Fig. S3E-F). IRF2 knockdown produce promotive effect on the apoptosis that can be reverted by introducing CENP-N in NPC cells (Fig. S3G). IRF2 knockdown produce suppressive effect on the G0/G1 phase progression that can be reverted by introducing CENP-N in NPC cells (Fig. S3H).

### CENP-N affects the glucose metabolism, proliferation, cell cycling and apoptosis of NPC cells in vitro and in vivo through the AKT pathway

**Fig. 7:**
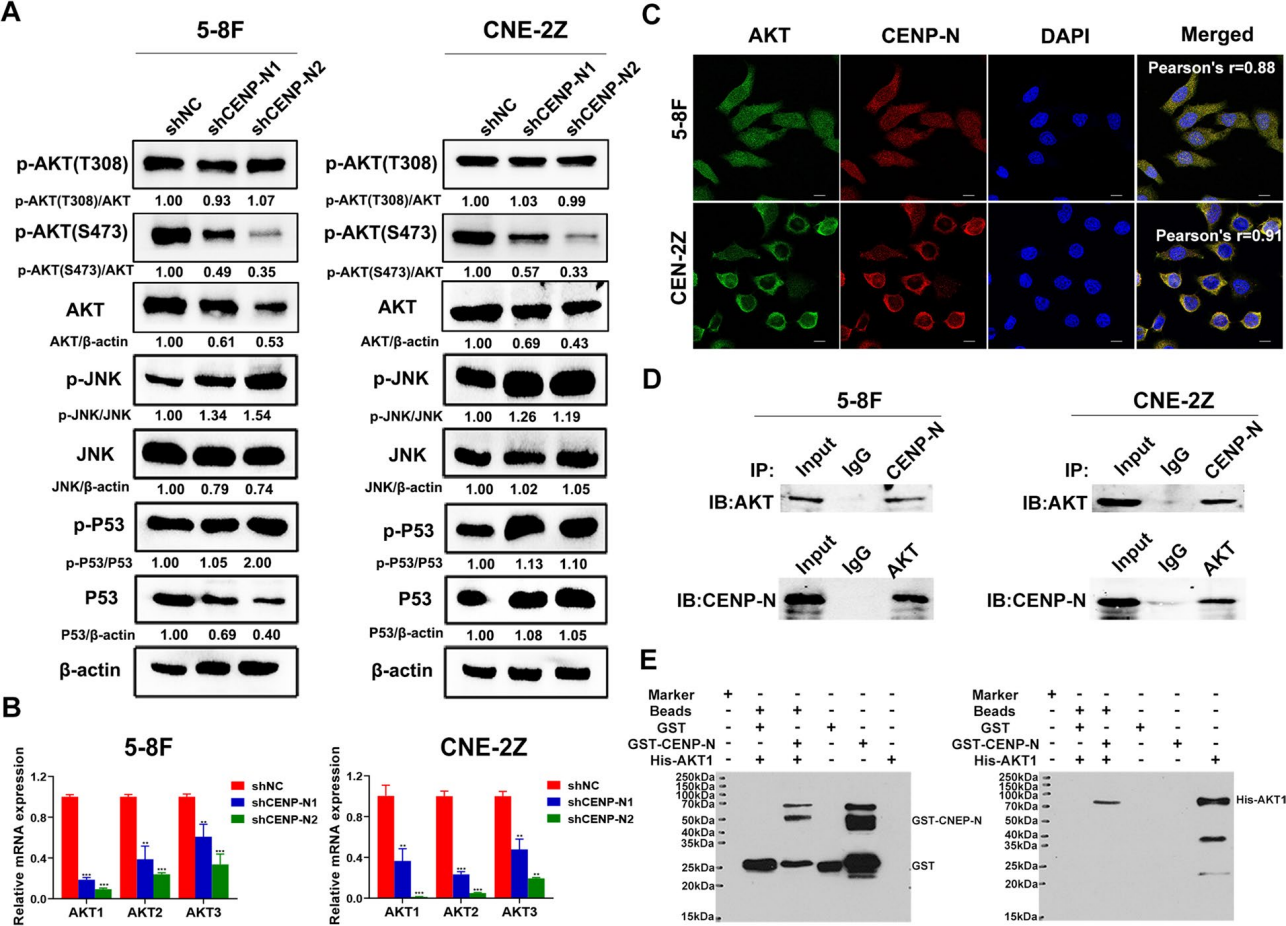
The tumor-promoting properties of CENP-N may be mediated via the AKT signaling pathway. **a** Changes in the expression of core proteins in the related signaling pathway after CENP-N knockdown in 5-8F and CNE-2Z cells. **b** Changes in AKT1, AKT2, and AKT3 mRNA expression after CENP-N knockdown in 5-8F and CNE-2Z cells. **c** An immunofluorescence assay was performed to detect the colocalization of CENP-N and AKT in 5-8F and CNE-2Z cells (scale bar, 10 μm). **d** The results of the coimmunoprecipitation assay demonstrated the interaction between CENP-N and AKT. **e** The results of the GST fusion protein pulldown assay demonstrated a direct interaction between CENP-N and AKT1. The data are shown as the mean ± SD values. ** *p* < 0.01, ***p < 0.001

To further investigate the molecular mechanisms involved in the regulation of malignant biological behaviors of NPC cells by CENP-N, we examined the changes in the expression and phosphorylation levels of core proteins in common signaling pathways involved in the regulation of glucose metabolism, cell proliferation, cell cycling and apoptosis. The results showed that relative to the levels in the shNC group, the levels of p-AKT (S473) and AKT were decreased and those of p-JNK and p-P53 were increased in both NPC cell lines after knockdown of CENP-N, while those of p-AKT (T308) and P53 were not significantly changed. The JNK was decreased in 5-8F cells, while it was not significantly changed in CNE-2Z cells (Fig. 7a). This result confirmed that the most significant change in the AKT signaling pathway was observed in NPC cells after knockdown of CENP-N and was mainly affected by phosphorylation at S473.

Subsequently, we found that the AKT1, AKT2, and AKT3 mRNA levels were decreased in the shCENP-N1 and shCENP-N2 groups relative to the shNC group (Fig. 7b). We also found a significant decrease in the level of p-AKT (S473) in the CENP-N knockdown group by immunofluorescence staining (Fig. S4A), immunohistochemistry (Fig. S4B) and Western blot analysis (Fig. S4C) in nude mouse tumor tissues (*P* < 0.05).

The cellular immunofluorescence staining results revealed that CENP-N colocalized with the AKT protein in 5-8F and CNE-2Z cells (Fig. 7c). This result suggested a protein-protein interaction between CENP-N and AKT might occur in NPC cells. Subsequently, we examined the protein-protein interaction of CENP-N with AKT by Co-IP assay in the NPC cell lines 5-8F and CNE-2Z (Fig. 7d). The protein-protein interaction of CENP-N with AKT1 was detected by a GST pulldown assay in recipient cells expressing the GST-CENP-N fusion protein and His-AKT1 fusion protein (Fig. 7e). These results revealed that there was a direct protein-protein interaction between CENP-N and AKT1 in these cells.

Next, we investigated whether the AKT inhibitor MK-2206 can block the oncogenic effect of overexpressed CENP-N on NPC cells. We also overexpressed CENP-N in 5-8F and CNE-2Z cells using a lentiviral vector (Fig. S5A). Subsequently, we examined the changes in the malignant biological behavior of the oeCENP-N and oeCENP-N + MK-2206 groups of NPC cells using oeControl cells as the control. The results showed that MK-2206 blocked the promotion of glucose metabolism in NPC cells induced by overexpression of CENP-N (Fig. S5B-C). MK-2206 blocked the promotion of the survival and proliferation of NPC cells induced by overexpression of CENP-N (Fig. S5D-E). MK-2206 blocked the inhibitory effect of CENP-N overexpression on the apoptosis of NPC cells (Fig. S5F). MK-2206 blocked the promotive effect of CENP-N overexpression on NPC cell cycling (Fig. S5G).

These results indicated that the down-regulation of CENP-N on NPC cells significantly inhibited AKT phosphorylation at Ser473, leading to the inhibition of AKT activation and downstream potential effects on AKT signaling.

### CENP-N promotes cell proliferation and cell cycling and inhibits apoptosis by promoting aerobic glycolysis in NPC cells

To understand whether CENP-N in 5-8F and CNE-2Z cells promotes cell proliferation and cell cycling and inhibits apoptosis by promoting glucose metabolism, we established three groups of NPC cells—oeControl, oeCENP-N and oeCENP-N + 2-DG (a glucose analog and an inhibitor of glucose metabolism)—and examined these three groups of cells. Glucose uptake and lactate production, cell proliferation, apoptosis and cell cycle changes were examined. The results showed that 2-DG blocked the promotion of glucose metabolism in NPC cells induced by overexpression of CENP-N (Fig. S6A-B). 2-DG blocked the promotion of NPC cell survival and proliferation induced by overexpression of CENP-N (Fig. S6C-D). 2-DG blocked the inhibitory effect of CENP-N overexpression on apoptosis (Fig. S6E). 2-DG blocked the promotive effect of CENP-N overexpression on NPC cell cycling (Fig. S6F).

**Fig. 8:**
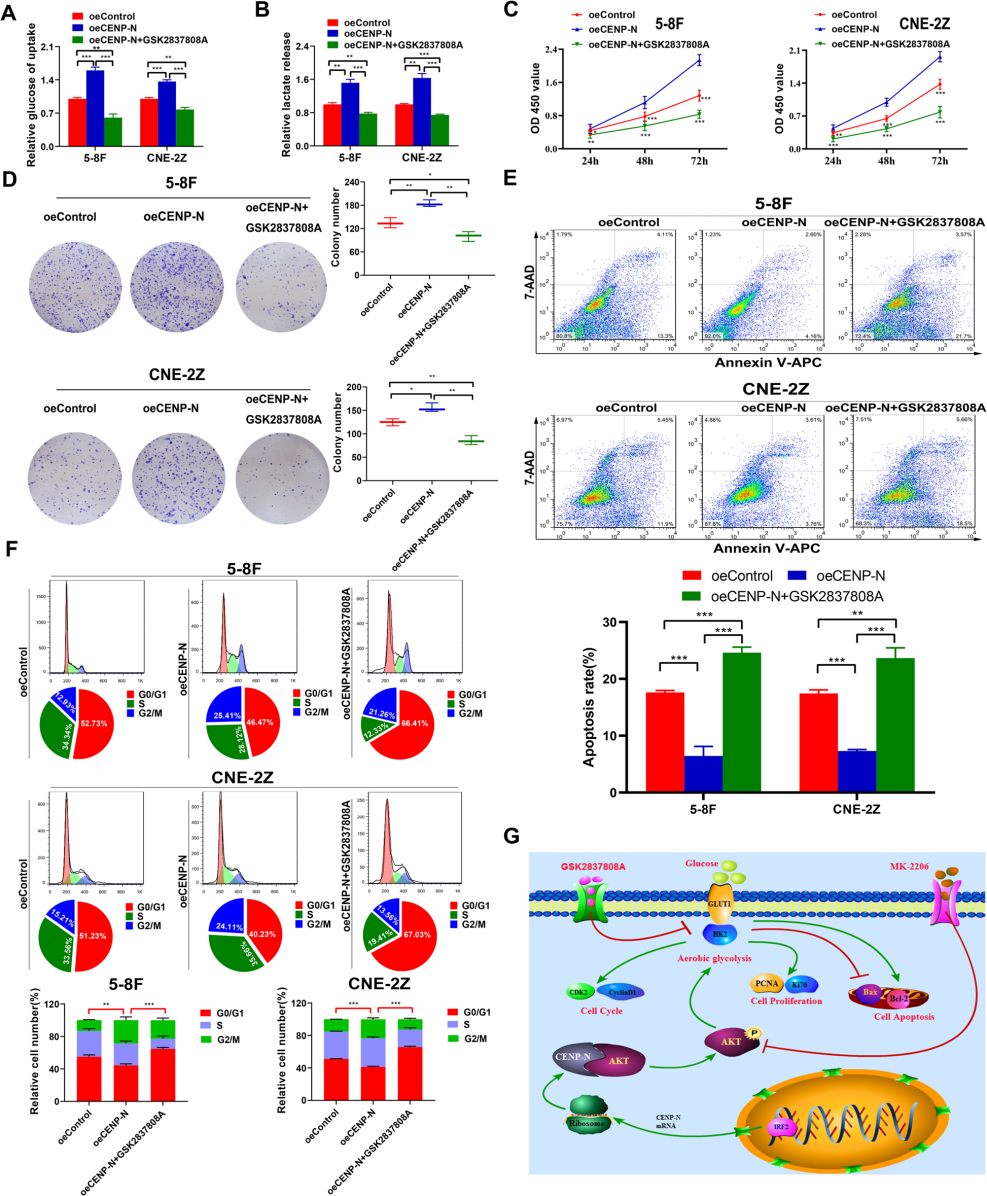
CENP-N promotes cell proliferation, cell cycling and apoptosis resistance by increasing aerobic glycolysis in NPC cells. **a** Changes in relative glucose uptake in two NPC cell lines were detected after CENP-N overexpression and GSK2837808A treatment. **b** Changes in lactic acid production in two NPC cell lines were detected after CENP-N overexpression and GSK2837808A treatment. **c** A CCK-8 assay was used to detect changes in cell viability after CENP-N overexpression and GSK2837808A treatment in two NPC cell lines. **d** Clone formation assay was used to detect the changes in cell proliferation after CENP-N overexpression and GSK2837808A treatment in two NPC cell lines. **e** The percentage of apoptotic cells was determined in two NPC cell lines after CENP-N overexpression and GSK2837808A treatment. **f** Changes in the cell cycle distribution in two NPC cell lines after CENP-N overexpression and GSK2837808A treatment. **g** Model showing the action mechanism of CENP-N in NPC. The data are shown as the mean ± SD values. * *p* < 0.05, ** *p* < 0.01, ***p < 0.001

However, 2-DG is a glucose uptake inhibitor. In order to investigate whether CENP-N promotes tumorigenesis mainly by promoting aerobic glucose utilization, we established three groups of NPC cells—oeControl, oeCENP-N and oeCENP-N + GSK2837808A (a specific inhibitor of LDHA)—and examined these three groups of cells. Glucose uptake and lactate production, cell proliferation, apoptosis and cell cycle changes were examined. The results showed that GSK2837808A blocked the promotion of aerobic glycolysis in NPC cells induced by overexpression of CENP-N (Fig. 8a-b). GSK2837808A blocked the promotion of NPC cell survival and proliferation induced by overexpression of CENP-N (Fig. 8c-d). GSK2837808A blocked the inhibitory effect of CENP-N overexpression on apoptosis (Fig. 8e). GSK2837808A blocked the promotive effect of CENP-N overexpression on NPC cell cycling(Fig. 8f)).

Taken together, the results of the present study confirmed the role of the IRF2/CENP-N/AKT signaling axis in promoting malignant biological behaviors of NPC cells in vitro and in vivo by increasing aerobic glycolysis (Fig. 8g).

## Discussion

This study first confirmed that CENP-N was highly expressed in NPC tissues and predicted a poor prognosis for patients with NPC, suggesting that CENP-N may be an oncogene in NPC. This result is consistent with the findings of Wang et al., who found high expression of CENP-N in breast cancer [35]. Interestingly, we found by staining tissue specimens from NPC patients combined with examining clinical PET/CT data that the glucose uptake of NPC tissues with high expression of CENP-N was also elevated; thus, we speculated that the expression of CENP-N in NPC affects cellular glucose metabolism.

Transcriptome sequencing has unique advantages in cancer diagnosis and prognostic assessment. The most commonly used second-generation sequencing studies currently are based on RNA sequence analysis to identify differentially expressed genes in order to understand the molecular mechanisms involved in various biological processes and thus guide cancer diagnosis or treatment [36]. We generated NPC cells with CENP-N knockdown based on clinical observations and subsequently performed transcriptome sequencing to analyze the differentially expressed genes in the CENP-N knockdown group of cells. The results revealed that knockdown of CENP-N resulted in significant changes in key genes related to cellular glucose metabolism, the cell cycle, and cell proliferation.

Zhang et al. found that inhibition of aerobic glycolysis in NPC cells significantly reduced the proliferation and invasion abilities of NPC cells [30], suggesting that aerobic glycolysis is one of the driving factors of NPC progression. In this study, experiments in vitro and in nude mice revealed that knockdown of CENP-N expression in NPC cell lines significantly reduced glucose uptake in NPC cells. These results, together with PET/CT data for NPC patients, confirmed that CENP-N is involved in the regulation of glucose metabolism in NPC cells, which is dominated by aerobic glycolysis.

The transcription factor can regulate the transcription of different genes in different cell types and thus can play different biological roles [37]. IRF2 can play different or even opposing roles in different tumors by participating in the regulation of different molecular biological mechanisms. Wang et al. found that overexpression of IRF2 promoted the proliferation of NT2 testicular embryonal carcinoma cells [38]. However, it has also been demonstrated that IRF2 acts as an oncogene in skin squamous and colorectal cancers [39, 40]. In the present study, we found that IRF2 in NPC cells bound directly to the CENP-N promoter sequence and promoted the transcription of CENP-N and that the expression of CENP-N was significantly downregulated after knocking down the expression of IRF2. Furthermore, we found that overexpression of CENP-N effectively reversed the inhibitory effects of IRF2 knockdown on glucose metabolism, proliferation, cell cycling and apoptosis resistance in NPC cells. This finding confirmed that IRF2 is an upstream transcription factor of CENP-N and can regulate a series of malignant biological behaviors of NPC cells through CENP-N.

Previous studies have reported that P53, AKT, and JNK, as core molecules of classical signaling pathways in NPC, are involved in regulating a variety of malignant biological behaviors in NPC cells [41–43]. In the present study, we found that among many factors, knockdown of CENP-N resulted in significant changes in the expression and phosphorylation of AKT and that the main changes occurred in the form of AKT phosphorylated at S473. It was reported that phosphorylation of AKT Thr308 by PDK1 and AKT Ser473 by mTORC2 could activate AKT [44]. Inhibition of PI3K could block AKT phosphorylation at Thr308, leading to decreased downstream signaling to mTORC1 [45]. However, PI3K/AKT/mTORC1 suppression relieves feedback inhibition to upstream network effectors, including mTORC2, causing a recovery of AKT signaling [46]. Therefore, the AKT Ser473 phosphorylation site may play a more important role in the activation of AKT and downstream potential effects on AKT signaling.

Our previous studies also confirmed that AKT is widely involved in the regulation of biological behaviors such as proliferation, cell cycling, apoptosis and invasion in NPC cells [24, 32]. In the present study, we found that AKT1, AKT2, and AKT3 expression was downregulated after knockdown of CENP-N in NPC cells. Therefore, we inferred that the effects of CENP-N on NPC cell behaviors might be related to AKT. Then, we found that the CENP-N and AKT proteins were colocalized and that a protein-protein interaction between CENP-N and AKT occurred in NPC cells both under physiological conditions and under exogenous expression conditions. Zhao et al. demonstrated that the AKT inhibitor MK-2206 effectively blocked AKT expression and phosphorylation in NPC cells [47]. In the present study, we found that the promotive effects of CENP-N overexpression on malignant biological behaviors of NPC cells were blocked after treatment with the AKT inhibitor MK-2206. Accordingly, this study confirmed that CENP-N can affect the biological behavior of NPC cells by regulating the phosphorylation level of AKT at S473 after direct binding to AKT in the cell.

A study by Cai et al. found that the use of siRNA to interfere with Chibby expression inhibited aerobic glycolysis and cell proliferation in NPC cells [48]. The results of this study showed that knockdown of CENP-N effectively inhibited glucose metabolism, cell proliferation, and cell cycle arrest and promoted apoptosis in NPC cell lines. Both the glucose uptake inhibitor 2-DG and the LDHA inhibitor GSK2837808A could block the promotion of proliferation, cell cycling and apoptosis resistance of NPC cells induced by overexpression of CENP-N. As we all know, aerobic glycolysis is the main mode of glucose metabolism in cancer cells [49]. Thus, we not only report for the first time that CENP-N expression is elevated in NPC and associated with poor patient prognosis but also confirm that CENP-N enhances cell proliferation, cell cycling and apoptosis resistance by promoting aerobic glycolysis in NPC cells.

At present, the combination of radiotherapy and chemotherapy is the first choice for the treatment of NPC. However, when patients relapse or develop distant metastases, these current treatments are less effective. It is urgent to develop new immunotherapies or small molecule targeted drugs to treat these patients. In this study, we found that CENP-N protein was mainly expressed in the cytoplasm of NPC cells. Since some chemosynthetic small-molecule drugs could enter the cytoplasm to inhibit BRAF mutation and treated melanoma [50], some small-molecule drugs that can specifically inhibit or degrade CENP-N could be designed to treat nasopharyngeal cancer in the future.

## Conclusions

To summarize, this study confirms that the IRF2/CENP-N/AKT signaling axis promotes proliferation, cell cycling and apoptosis resistance in NPC cells by affecting aerobic glycolysis. This signaling axis is expected to be a new therapeutic target for NPC.

## Supplementary Information

**Additional file 1: Supplementary Figure 1.** Bioinformatic analysis of differentially expressed genes and prognosis in NPC. a Volcano plot showing differentially expressed genes in the GSE12452 and GSE53819 microarray datasets (https://www.ncbi.nlm.nih.gov/geo/). b Venn diagram showing the intersection of up- and downregulated genes in the GSE12452 and GSE53819 microarray datasets. c PPI networks associated with the differentially expressed genes. d Kaplan-Meier survival curves for patients stratified by the expression levels of differentially expressed genes (http://kmplot.com/analysis/). e Disease-free survival curves for HNSCC patients stratified by the expression levels of core genes (http://gepia.cancer-pku.cn/). f Histogram showing CENP-N expression in normal tissues (https://www.ncbi.nlm.nih.gov/gene/). g Linear plot of CENP-N expression in a pancancer dataset (http://gepia.cancer-pku.cn/).

**Additional file 2: Supplementary Figure 2.** In vivo experimental immunohistochemical analysis. a HE staining of all four groups of tumors (scale bar, 50 μm). b Immunohistochemical analysis of in vivo glucose metabolism-, cell proliferation-, cell cycle-, and apoptosis-related protein expression in tumors (scale bar, 50 μm). c Statistical analysis of the immunohistochemical results for in vivo glucose metabolism-, cell proliferation-, cell cycle-, and apoptosis-related protein expression in tumors (IOD, the cumulative optical density). The data are expressed as the mean ± SD values. * *p* < 0.05, ** *p* < 0.01, ***p < 0.001.

**Additional file 3: Supplementary Figure 3.** Overexpression of CENP-N blocks the effect of IRF2 downregulation on malignant biological behaviors of NPC cells. a Combined with venn diagram, five transcription factors (IRF2, YY1, WT1, ATF2 and ELK1) were shared as the transcription factors of CENP-N. b Expression of IRF2 in NPC and NP460 cell lines. c Changes in relative cellular glucose uptake detected after downregulation of IRF2 and overexpression of CENP-N in two NPC cell lines. d Changes in cellular lactate production detected after downregulation of IRF2 and overexpression of CENP-N in two NPC cell lines. e Changes in cell viability detected by a CCK-8 assay after downregulation of IRF2 and overexpression of CENP-N in two NPC cell lines. f Changes in cell proliferation detected by a colony formation assay after downregulation of IRF2 and overexpression of CENP-N in two NPC cell lines. g Changes in apoptosis detected after downregulation of IRF2 and overexpression of CENP-N in two NPC cell lines. h Changes in the cell cycle distribution after downregulation of IRF2 and overexpression of CENP-N in 5-8F and CNE-2Z cell lines. The data are expressed as the mean ± SD values. * *p* < 0.05, ** *p* < 0.01, ***p < 0.001.

**Additional file 4: Supplementary Figure 4.** Changes in AKT S473 phosphorylation in nude mouse tumor tissues. a p-AKT (S473) immunofluorescence staining in tumor tissue in vivo (scale bar, 50 μm). b p-AKT (S473) immunohistochemical staining in tumor tissue in vivo (scale bar, 50 μm). c p-AKT (S473) protein expression in tumor tissue in vivo was detected using WB.

**Additional file 5: Supplementary Figure 5.** AKT inhibitors block the effect of CENP-N overexpression on malignant biological behaviors of NPC cells. a Changes in the relative cellular glucose uptake after overexpression of CENP-N or treatment with an AKT inhibitor in two NPC cell lines. b Changes in cellular lactate production after overexpression of CENP-N or treatment with an AKT inhibitor in two NPC cell lines. c Changes in the viability of cells as detected by a CCK-8 assay after overexpression of CENP-N or treatment with an AKT inhibitor in two NPC cell lines. d Changes in the proliferation capacity of cells as detected by a colony formation assay after overexpression of CENP-N or treatment with an AKT inhibitor in two NPC cell lines. e Changes in the percentage of apoptotic cells detected after overexpression of CENP-N or treatment with an AKT inhibitor in two NPC cell lines. f Changes in the cell cycle distribution after overexpression of CENP-N or treatment with an AKT inhibitor in 5-8F and CNE-2Z cell lines. The data are expressed as the mean ± SD values. * *p* < 0.05, ** *p* < 0.01, ***p < 0.001.

**Additional file 6: Supplementary Figure 6.** CENP-N promotes cell proliferation, cell cycling and apoptosis by increasing glucose metabolism in NPC cells. a Changes in relative glucose uptake in two NPC cell lines were detected after CENP-N overexpression and 2-DG treatment. b Changes in lactic acid production in two NPC cell lines were detected after CENP-N overexpression and 2-DG treatment. c A CCK-8 assay was used to detect changes in cell viability after CENP-N overexpression and 2-DG treatment in two NPC cell lines. d Clone formation assay was used to detect the changes in cell proliferation after CENP-N overexpression and 2-DG treatment in two NPC cell lines. e The percentage of apoptotic cells was determined in two NPC cell lines after CENP-N overexpression and 2-DG treatment. f Changes in the cell cycle distribution in two NPC cell lines after CENP-N overexpression and 2-DG treatment. The data are shown as the mean ± SD values. * *p* < 0.05, ** *p* < 0.01, ***p < 0.001.

**Additional file 7: Supplementary Table 1.** The website addresses of datasets. **Supplementary Table 2.** The general characteristics of NPC patients with PET/CT imaging data. **Supplementary Table 3.** The references for all antibodies used. **Supplementary Table 4.** List of primers used for qRT-PCR analysis. **Supplementary Table 5**. List of primers used for ChIP analysis. **Supplementary Table 6.** Tissue microarray analysis of CENP-N expression.

## Data Availability

The authors declare that all data and materials are available on request.

## Abbreviations

NPC: Nasopharyngeal carcinoma
NPG: Nasopharyngitis tissue
CENP-N: Centromere protein N
LDHA: Lactate dehydrogenase A
HNSCC: Head and neck squamous cell carcinoma
ChIP: Chromatin immunoprecipitation
Co-IP: Co-immunoprecipitation
2-DG: 2-deoxy-d-glucose
IRF2: Interferon regulatory factor 2
AKT: Protein kinase B
DEGs: Differentially expressed genes
GEO: Gene Expression Omnibus
PPI: Protein protein interaction
KSFM: Keratinocyte serum-free medium
TMA: Tissue microarray
OD: Optical density
CCK-8: Cell Counting Kit-8
qRT-PCR: Quantitative real-time PCR
KEGG: Kyoto Encyclopedia of Genes and Genomes
SUVmax: The maximum standardized uptake value
GO: Gene Ontology
BP: Biological process
CC: Cell component
MF: Molecular function
SUVmean: The mean standardized uptake value
TLG: Total lesion glycolysis
shCENP-N: Small hairpin RNA expression vector targeting human CENP-N gene
shNC: Negative control with small hairpin RNA expression vector
TNM stage: Tumor-node-metastasis stage
