# Supplementary figures and images for: The IRF2/CENP-N/AKT signaling axis promotes proliferation, cell cycling and apoptosis resistance in nasopharyngeal carcinoma cells by increasing aerobic glycolysis
Source: J Exp Clin Cancer Res. 2021 Dec 10;40:390. doi: 10.1186/s13046-021-02191-3 (PMC8662847; doi:10.1186/s13046-021-02191-3)

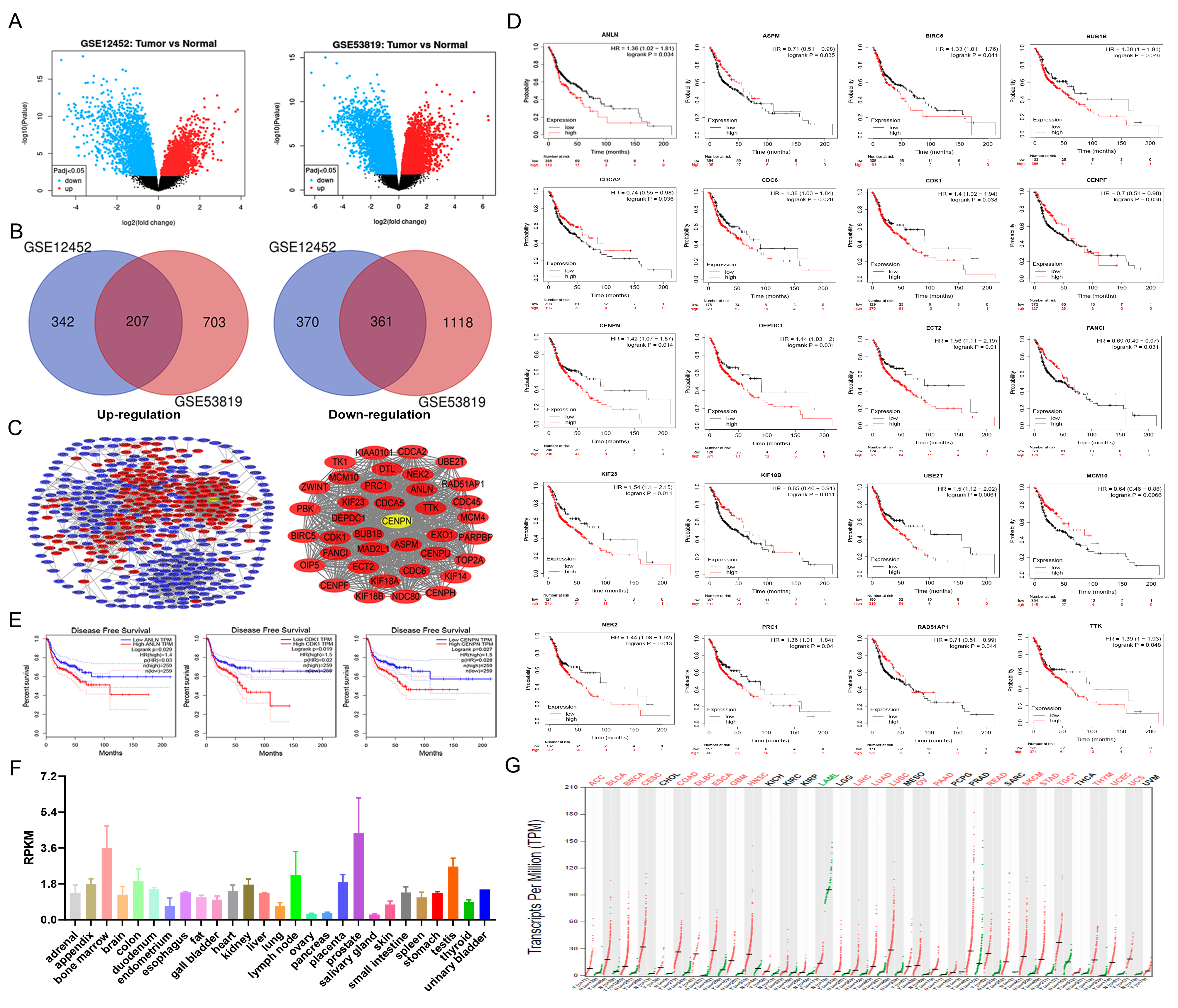

Supplement: Supplementary file 1 — Additional file 1: Supplementary Figure 1. Bioinformatic analysis of differentially expressed genes and prognosis in NPC. a Volcano plot showing differentially expressed genes in the GSE12452 and GSE53819 microarray datasets (https://www.ncbi.nlm.nih.gov/geo/). b Venn diagram showing the intersection of up- and downregulated genes in the GSE12452 and GSE53819 microarray datasets. c PPI networks associated with the differentially expressed genes. d Kaplan-Meier survival curves for patients stratified by the expression levels of differentially expressed genes (http://kmplot.com/analysis/). e Disease-free survival curves for HNSCC patients stratified by the expression levels of core genes (http://gepia.cancer-pku.cn/). f Histogram showing CENP-N expression in normal tissues (https://www.ncbi.nlm.nih.gov/gene/). g Linear plot of CENP-N expression in a pancancer dataset (http://gepia.cancer-pku.cn/). [file 13046_2021_2191_MOESM1_ESM.tif]

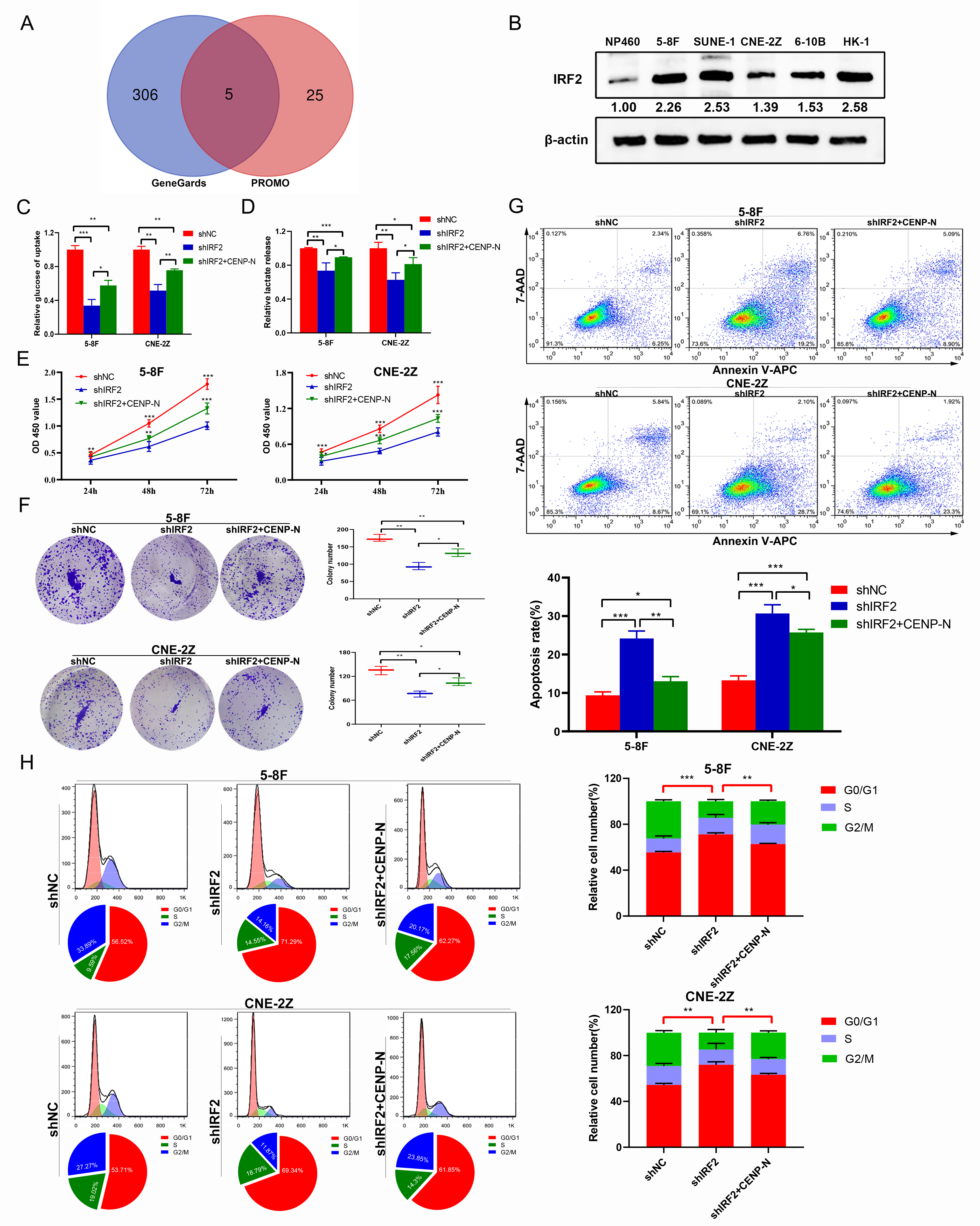

Supplement: Supplementary file 3 — Additional file 3: Supplementary Figure 3. Overexpression of CENP-N blocks the effect of IRF2 downregulation on malignant biological behaviors of NPC cells. a Combined with venn diagram, five transcription factors (IRF2, YY1, WT1, ATF2 and ELK1) were shared as the transcription factors of CENP-N. b Expression of IRF2 in NPC and NP460 cell lines. c Changes in relative cellular glucose uptake detected after downregulation of IRF2 and overexpression of CENP-N in two NPC cell lines. d Changes in cellular lactate production detected after downregulation of IRF2 and overexpression of CENP-N in two NPC cell lines. e Changes in cell viability detected by a CCK-8 assay after downregulation of IRF2 and overexpression of CENP-N in two NPC cell lines. f Changes in cell proliferation detected by a colony formation assay after downregulation of IRF2 and overexpression of CENP-N in two NPC cell lines. g Changes in apoptosis detected after downregulation of IRF2 and overexpression of CENP-N in two NPC cell lines. h Changes in the cell cycle distribution after downregulation of IRF2 and overexpression of CENP-N in 5-8F and CNE-2Z cell lines. The data are expressed as the mean ± SD values. * p < 0.05, ** p < 0.01, ***p < 0.001. [file 13046_2021_2191_MOESM3_ESM.tif]

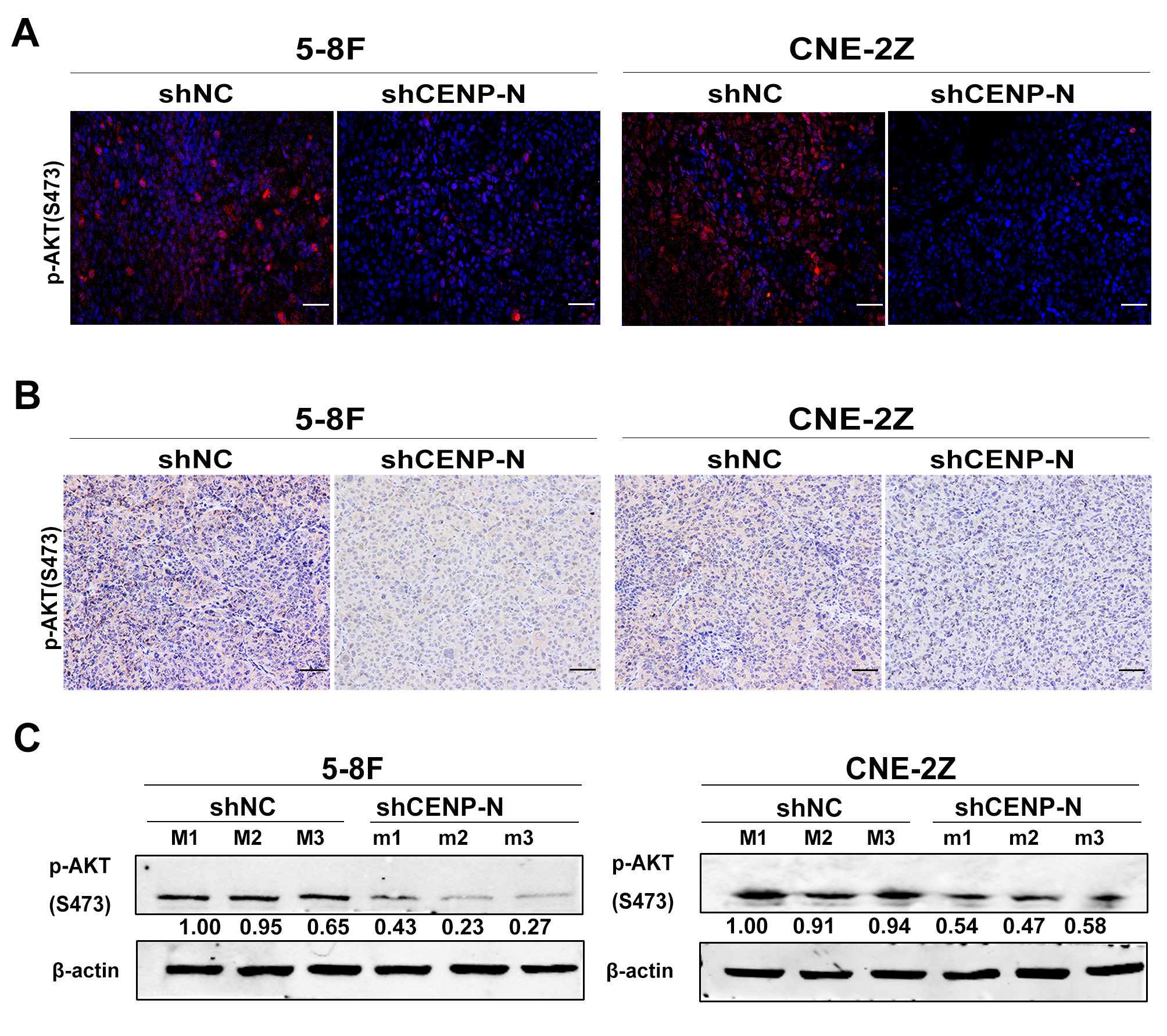

Supplement: Supplementary file 4 — Additional file 4: Supplementary Figure 4. Changes in AKT S473 phosphorylation in nude mouse tumor tissues. a p-AKT (S473) immunofluorescence staining in tumor tissue in vivo (scale bar, 50 μm). b p-AKT (S473) immunohistochemical staining in tumor tissue in vivo (scale bar, 50 μm). c p-AKT (S473) protein expression in tumor tissue in vivo was detected using WB. [file 13046_2021_2191_MOESM4_ESM.tif]

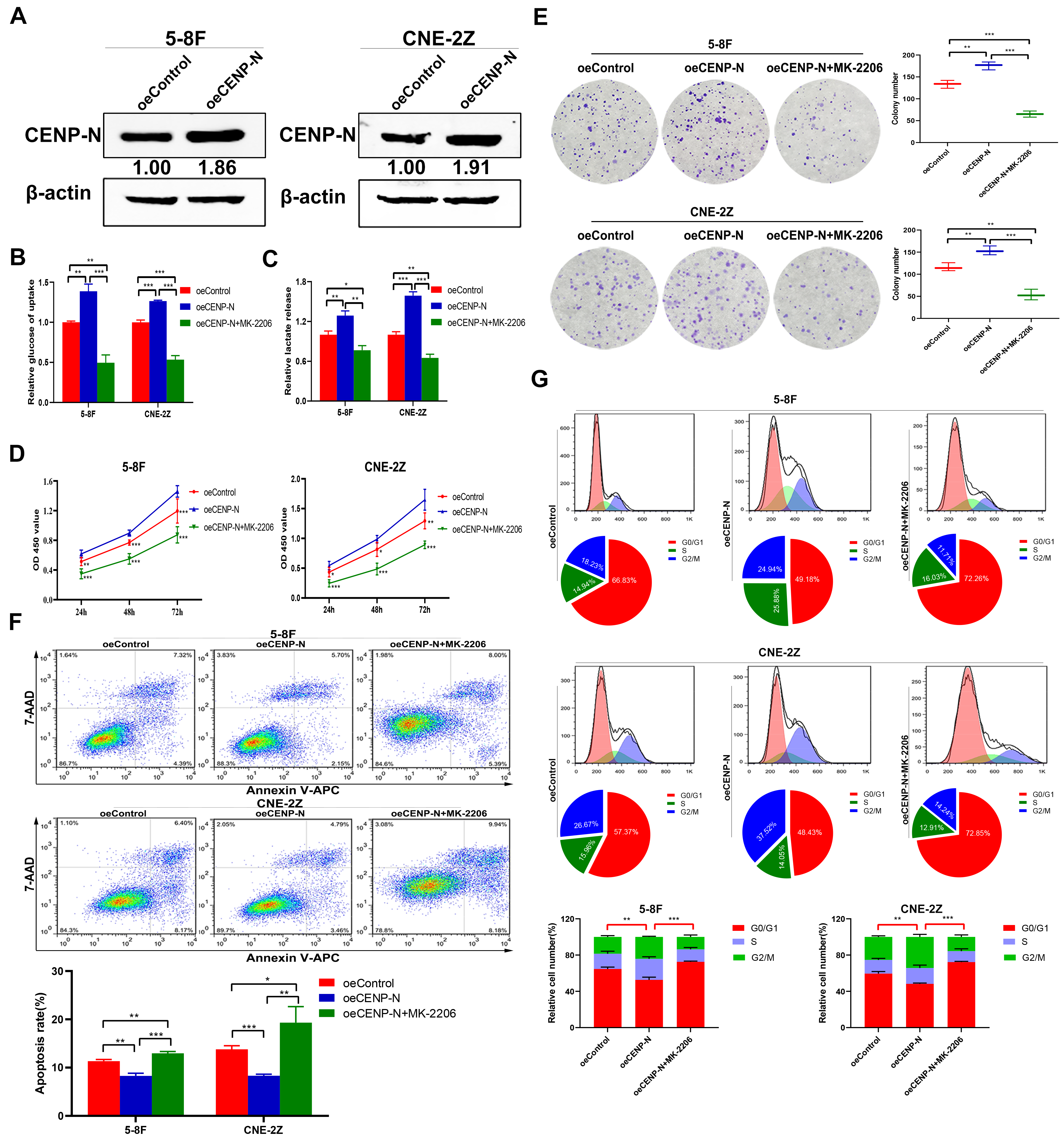

Supplement: Supplementary file 5 — Additional file 5: Supplementary Figure 5. AKT inhibitors block the effect of CENP-N overexpression on malignant biological behaviors of NPC cells. a Changes in the relative cellular glucose uptake after overexpression of CENP-N or treatment with an AKT inhibitor in two NPC cell lines. b Changes in cellular lactate production after overexpression of CENP-N or treatment with an AKT inhibitor in two NPC cell lines. c Changes in the viability of cells as detected by a CCK-8 assay after overexpression of CENP-N or treatment with an AKT inhibitor in two NPC cell lines. d Changes in the proliferation capacity of cells as detected by a colony formation assay after overexpression of CENP-N or treatment with an AKT inhibitor in two NPC cell lines. e Changes in the percentage of apoptotic cells detected after overexpression of CENP-N or treatment with an AKT inhibitor in two NPC cell lines. f Changes in the cell cycle distribution after overexpression of CENP-N or treatment with an AKT inhibitor in 5-8F and CNE-2Z cell lines. The data are expressed as the mean ± SD values. * p < 0.05, ** p < 0.01, ***p < 0.001. [file 13046_2021_2191_MOESM5_ESM.tif]

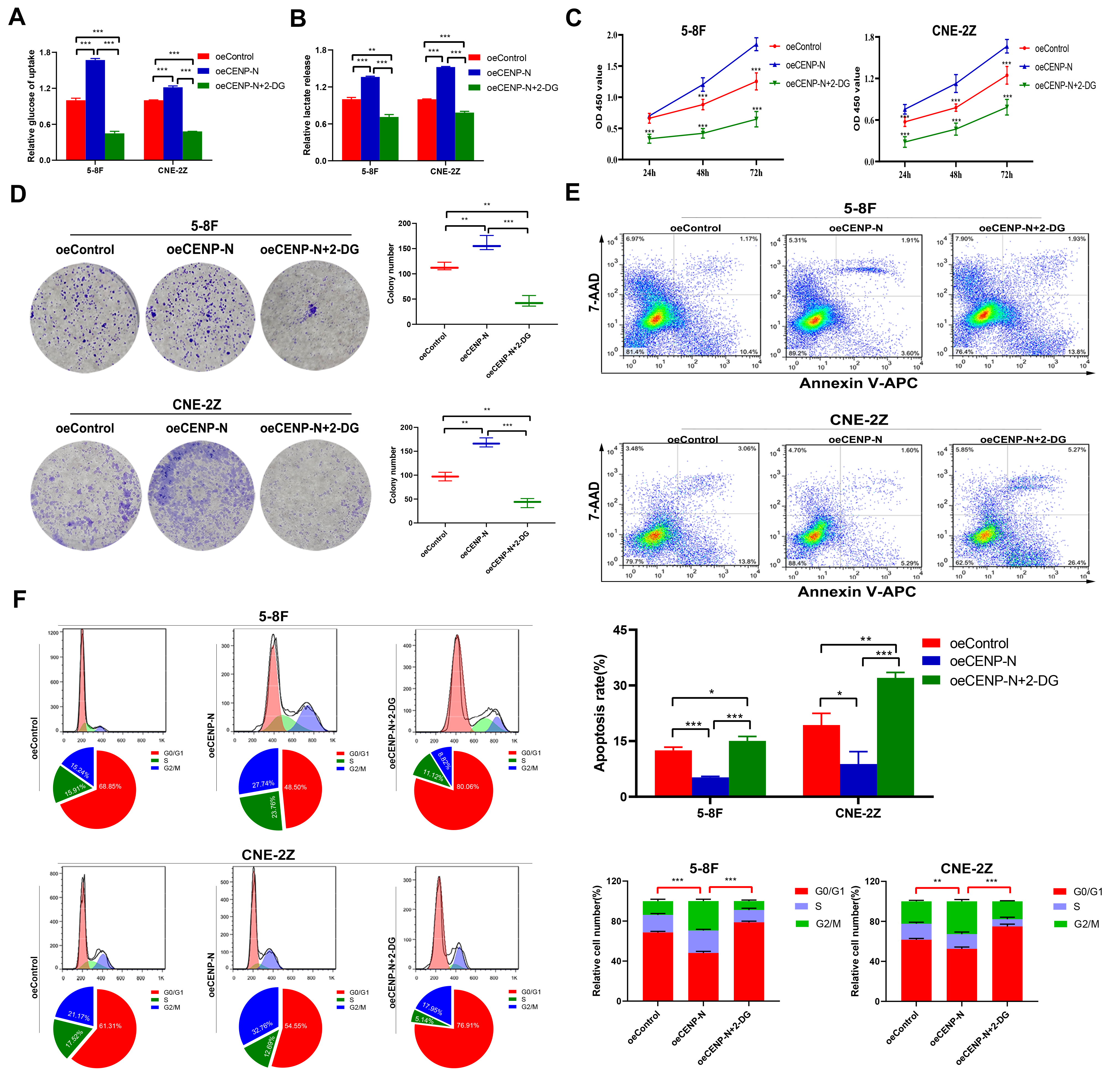

Supplement: Supplementary file 6 — Additional file 6: Supplementary Figure 6. CENP-N promotes cell proliferation, cell cycling and apoptosis by increasing glucose metabolism in NPC cells. a Changes in relative glucose uptake in two NPC cell lines were detected after CENP-N overexpression and 2-DG treatment. b Changes in lactic acid production in two NPC cell lines were detected after CENP-N overexpression and 2-DG treatment. c A CCK-8 assay was used to detect changes in cell viability after CENP-N overexpression and 2-DG treatment in two NPC cell lines. d Clone formation assay was used to detect the changes in cell proliferation after CENP-N overexpression and 2-DG treatment in two NPC cell lines. e The percentage of apoptotic cells was determined in two NPC cell lines after CENP-N overexpression and 2-DG treatment. f Changes in the cell cycle distribution in two NPC cell lines after CENP-N overexpression and 2-DG treatment. The data are shown as the mean ± SD values. * p < 0.05, ** p < 0.01, ***p < 0.001. [file 13046_2021_2191_MOESM6_ESM.tif]
