# Supplementary material for: The IRF2/CENP-N/AKT signaling axis promotes proliferation, cell cycling and apoptosis resistance in nasopharyngeal carcinoma cells by increasing aerobic glycolysis
Source: J Exp Clin Cancer Res. 2021 Dec 10;40:390. doi: 10.1186/s13046-021-02191-3 (PMC8662847; doi:10.1186/s13046-021-02191-3)
Supplement: Supplementary file 7 — Additional file 7: Supplementary Table 1. The website addresses of datasets. Supplementary Table 2. The general characteristics of NPC patients with PET/CT imaging data. Supplementary Table 3. The references for all antibodies used. Supplementary Table 4. List of primers used for qRT-PCR analysis. Supplementary Table 5. List of primers used for ChIP analysis. Supplementary Table 6. Tissue microarray analysis of CENP-N expression. [file 13046_2021_2191_MOESM7_ESM.docx]

**Supplementary Table 1. The website addresses of datasets**

| Dataset | Web Link |
| --- | --- |
| GEO | <https://www.ncbi.nlm.nih.gov/geo/> |
| STRING | <http://string.embl.de/> |
| Kaplan–Meier plotter | <http://kmplot.com/analysis/> |
| GEPIA  GENE | <http://gepia.cancer-pku.cn/>  https://www.ncbi.nlm.nih.gov/gene/ |
| GeneCards  PROMO | <http://www.genecards.org/>  http://alggen.lsi.upc.es/ |
| JASPER | http://jaspar.genereg.net/ |
| GEPIA  KEGG  GO | <http://gepia.cancer-pku.cn/>  <http://www.genome.jp/kegg/>  <http://www.geneontology.org> |

| **Supplementary Table 2. The general characteristics of NPC**  **patients with PET/CT imaging data** | | | | | |
| --- | --- | --- | --- | --- | --- |
| Patient number | Gender | Age(y) | TNM Stage | CENP-N expression | 18F-FDG SUVmax |
| 1 | Male | 56 | T1N1M1 | Low | 2.1 |
| 2 | Male | 77 | T1N0M1 | Low | 2.1 |
| 3 | Male | 55 | T1N2M0 | Low | 2.2 |
| 4 | Male | 45 | T1N1M0 | Low | 3.5 |
| 5 | Male | 64 | T2N2M0 | Low | 4.3 |
| 6 | Male | 55 | T1N2M0 | Low | 4.4 |
| 7 | Male | 46 | T2N1M0 | Low | 4.7 |
| 8 | Male | 50 | T2N2M0 | Low | 7 |
| 9 | Male | 39 | T1N2M0 | Low | 16.7 |
| 10 | Male | 50 | T1N2M0 | High | 2.2 |
| 11 | Male | 48 | T1N0M0 | High | 5.8 |
| 12 | Male | 67 | T1N0M0 | High | 7.1 |
| 13 | Male | 62 | T3N2M0 | High | 7.6 |
| 14 | Male | 59 | T2N1M0 | High | 8.1 |
| 15 | Female | 62 | T3N1M0 | High | 10.9 |
| 16 | Female | 43 | T3N1M0 | High | 11.2 |
| 17 | Male | 14 | T2N2M1 | High | 14.5 |
| 18 | Female | 46 | T2N1M0 | High | 17.6 |
| 19 | Male | 46 | T3N2M0 | High | 18.3 |
| 20 | Male | 30 | T2N1M0 | High | 22.5 |
| 21 | Male | 38 | T2N3M0 | High | 23.6 |

Note: The pathological diagnosis of all NPC patients was nonkeratinizing undifferentiated carcinoma. In this study, patients with negative and weakly positive immunohistochemical scores for CENP-N were as assigned to the low expression group (IHC score≤3), and those with positive and strongly positive immunohistochemical scores for CENP-N were assigned to the high expression group (IHC score≥4).

**Supplementary Table 3. The references for all antibodies used**

| **Antibodies** | **Company** | **Number** | **Country** | **Applications** |
| --- | --- | --- | --- | --- |
| HK2 | Cell Signaling Technology^a^ | #2106 | USA | WB,IHC |
| GLUT1 | Cell Signaling Technology^a^ | #73015 | USA | WB,IHC |
| Ki67 | Cell Signaling Technology^a^ | #9129 | USA | WB,IHC |
| PCNA | Cell Signaling Technology^a^ | #13110 | USA | WB,IHC |
| Bax | Cell Signaling Technology^a^ | #5023 | USA | WB,IHC |
| Bcl-2 | Cell Signaling Technology^a^ | #4223 | USA | WB,IHC |
| CyclinD1 | Cell Signaling Technology^a^ | #55506 | USA | WB,IHC |
| CDK2 | Cell Signaling Technology^a^ | #18048 | USA | WB,IHC |
| p-JNK | Cell Signaling Technology^a^ | #9251 | USA | WB,IHC |
| JNK | Cell Signaling Technology^a^ | #9252 | USA | WB |
| p-P53 | Cell Signaling Technology^a^ | #2521 | USA | WB |
| P53 | Cell Signaling Technology^a^ | #2527 | USA | WB |
| p-AKT (T308) | Cell Signaling Technology^a^ | #13038 | USA | WB |
| p-AKT  (S473) | Cell Signaling Technology^a^ | #4060 | USA | WB,IHC |
| AKT1 | Cell Signaling Technology^a^ | #2938 | USA | WB,IP |
| AKT | Proteintech^b^ | 10176-2-AP | USA | IF |
| β-actin | Proteintech^b^ | 20536-1-AP | USA | WB |
| IRF2 | Proteintech^b^ | 12525-1-AP | USA | WB,IHC |
| IRF2 | Abcame^c^ | ab245658 | England | ChIP |
| CENP-N | Proteintech^d^ | 16751-1-AP | USA | WB,IHC |
| CENP-N | Novus^e^ | H00055839-PW1 | USA | IP |
| CENP-N | Novus^e^ | NBP1-79664 | USA | IF |
| Secondary Antibody | Licor^f^ | 926-68071 | USA | WB |

Note: ^a^<https://www.cellsignal.cn/>, ^b^<https://www.ptgcn.com/>, ^c^<https://www.abcam.com/>, ^d^https://www. ptgcn.com/, ^e^[https://www.novusbio.com/](file:///C:\Users\DCH\Documents\temp\htt,ps:\www.novusbio.com\),^f^ https://www.licor.com/, WB: western blot analysis, IP: immunoprecipitation, IHC: Immunohistochemical staining analysis, ChIP: Chromatin immunoprecipitation, IF: immunofluorescence.

**Supplementary Table 4. List of primers used for qRT-PCR analysis**

| Genes | Primer | Primer sequence(5’–3’) |
| --- | --- | --- |
| CENP-N | Forward primer | CACAAAGCCAAACCAGTACAAAC |
|  | Reverse primer | GATACCGACTTCTCAGGTCCATT |
| GLUT1 | Forward primer | GCTTCTCCAACTGGACCTCAAA |
|  | Reverse primer | GAAGAACAGAACCAGGAGCACAG |
| ENO1 | Forward primer | TGGAGCAGAGGTTTACCACAAC |
|  | Reverse primer | CGATAGACACCACTGGGTAGTCC |
| HK2 | Forward primer | GACTTCCGCACAGAATTTGATG |
|  | Reverse primer | GAATGTTACGGACAATCTCACCC |
| PFKFB2 | Forward primer | TTCTTTGTGGAATCCGTCTGTG |
|  | Reverse primer | AGGTCGGTAGGTAACTTTGTAGCA |
| PFKFB3 | Forward primer | CGACAAATGCGACAGGGACT |
|  | Reverse primer | CCTGGAGGTTGTGCTCGTTCT |
| LDHA | Forward primer | GATTCAGCCCGATTCCGTTAC |
|  | Reverse primer | GAGTCCAATAGCCCAGGATGTG |
| PKM2 | Forward primer | GGGAGCATTATGTCACCGGA |
|  | Reverse primer | CAGATGATGCCAGTGTTCCG |
| Ki67 | Forward primer | ACGCCTGGTTACTATCAAAAGG |
|  | Reverse primer | CAGACCCATTTACTTGTGTTGGA |
| PCNA | Forward primer | GAGTGGTCGTTGTCTTTC |
|  | Reverse primer | GCGGCAACAACGCCGCTA |
| c-MYC | Forward primer | GGAGGAACAAGAAGATGAGGAAG |
|  | Reverse primer | GCTGTGAGGAGGTTTGCTGTG |
| Bax | Forward primer | CGGGTTGTCGCCCTTTTCTA |
|  | Reverse primer | GAGGAAGTCCAATGTCCAGCC |
| Bcl-2 | Forward primer | GGAGGATTGTGGCCTTCTTTG |
|  | Reverse primer | GCATCCCAGCCTCCGTTATC |
| Caspase-3 | Forward primer | AGAACTGGACTGTGGCATTGAG |
|  | Reverse primer | CACAAAGCGACTGGATGAACC |
| Cyclin E1 | Forward primer | CACCTGACAAAGAAGATGATGACC |
|  | Reverse primer | AAGAGGGTGTTGCTCAAGAAAGT |
| Cyclin D1 | Forward primer | ATGCCAACCTCCTCAACGAC |
|  | Reverse primer | CTCCTCGCACTTCTGTTCCTC |
| CDK2 | Forward primer | TGCTCTCACTGGCATTCCTC |
|  | Reverse primer | TGGAGGACCCGATGAGAATG |
| AKT1 | Forward primer | TGGACTACCTGCACTCGGAGAA |
|  | Reverse primer | GTGCCGCAAAAGGTCTTCATGG |
| AKT2 | Forward primer | ACCCAACACCTTTGTCATACGC |
|  | Reverse primer | CACTTTAGCCCGTGCCTTG |
| AKT3 | Forward primer | CGGAAAGATTGTGTACCGTGATC |
|  | Reverse primer | CTTCATGGTGGCTGCATCTGTG |
| β-actin | Forward primer | CACCCAGCACAATGAAGATCAAGAT |
|  | Reverse primer | CCAGTTTTTAAATCCTGAGTCAAGC |

**Supplementary Table 5. List of primers used for ChIP analysis**

| Promoter site | Primer | Primer sequence(5’–3’) |
| --- | --- | --- |
| CENP-N1-1 | Forward primer | ATTACCAGGACTTACAAGGTGC |
|  | Reverse primer | TGCCATTCGTATCTCCTCTCC |
| CENP-N1-2 | Forward primer | TTACCAGGACTTACAAGGTGC |
|  | Reverse primer | TGCCATTCGTATCTCCTCTC |
| CENP-N1-3 | Forward primer | TACCAGGACTTACAAGGTGC |
|  | Reverse primer | GCCATTCGTATCTCCTCTCC |
| CENP-N2-1 | Forward primer | AAAGGAGAGGAGATACGAATGG |
|  | Reverse primer | GGCCGTTCAAGCAAGTCTC |
| CENP-N2-2 | Forward primer | AAAGGAGAGGAGATACGAATGG |
|  | Reverse primer | GCCGTTCAAGCAAGTCTCAG |
| CENP-N2-3 | Forward primer | AAAGGAGAGGAGATACGAATGG |
|  | Reverse primer | CGTTCAAGCAAGTCTCAGC |

**Supplementary Table 6. Tissue microarray analysis of CENP-N expression**

| Group | CENP-N expression | | χ^2^ | P value |
| --- | --- | --- | --- | --- |
|  | Low | High |  |  |
| NPG | 21 | 27 | 22.325 | 0.00*** |
| NPC | 20 | 138 |  |  |

Note: ***, P< 0.001.
